# Health, not weight loss, focused programmes versus conventional weight loss programmes for cardiovascular risk factors: a systematic review and meta-analysis

**DOI:** 10.1186/s13643-019-1083-8

**Published:** 2019-08-10

**Authors:** Nazanin Khasteganan, Deborah Lycett, Gill Furze, Andy P. Turner

**Affiliations:** Faculty of Health and Life Sciences, Coventry University, Coventry, UK

**Keywords:** Obesity, Weight loss, Systematic review, Meta-analysis, Non-diet, Intuitive eating, Cardiovascular disease, Well-being, Disordered eating behaviour

## Abstract

**Background:**

Obesity is a cardiovascular disease risk factor. Conventional weight loss (CWL) programmes focus on weight loss, however ‘health, not weight loss, focused’ (HNWL) programmes concentrate on improved health and well-being, irrespective of weight loss. What are the differences in CVD risk outcomes between these programmes?

**Aim:**

To conduct a systematic review and meta-analysis to compare the effects of HNWL with CWL programmes on cardiovascular disease risk factors.

**Methods:**

We searched CENTRAL, MEDLINE, EMBASE, PsycINFO, CINAHL, ASSIA, clinical trial registers, commercial websites and reference lists for randomised controlled trials comparing the two programmes (initially searched up to August 2015 and searched updated to 5 April 2019). We used the Mantel-Haneszel fixed-effect model to pool results. Sub-group and sensitivity analyses that accounted for variations in length of follow-up, enhanced programmes and risk of bias dealt with heterogeneity.

**Results:**

Eight randomised controlled trials of 20,242 potential studies were included. Improvements in total cholesterol-HDL ratio (mean difference − 0.21 mmol/L, 95% confidence interval [− 3.91, 3.50]) and weight loss (− 0.28 kg [− 2.00, 1.44]) favoured HNWL compared to CWL programmes in the long term (53–104 week follow-up), whereas improvements in systolic (− 1.14 mmHg, [− 5.84, 3.56]) and diastolic (− 0.15 mmHg, [− 3.64, 3.34]) blood pressure favoured CWL programmes. These differences did not reach statistical significance.

Statistically significant improvements in body satisfaction (− 4.30 [− 8.32, − 0.28]) and restrained eating behaviour (− 4.30 [− 6.77, − 1.83]) favoured HNWL over CWL programmes.

**Conclusions:**

We found no long-term significant differences in improved CVD risk factors; however, body satisfaction and restrained eating behaviour improved more with HNWL compared to CWL programmes. Yet firm conclusions cannot be drawn from small studies with high losses to follow-up and data sometimes arising from a single small study.

**Systematic review registration:**

PROSPERO CRD42015019505

**Electronic supplementary material:**

The online version of this article (10.1186/s13643-019-1083-8) contains supplementary material, which is available to authorized users.

## Background

Cardiovascular disease (CVD) is the leading cause of death across the globe [1]. In more than 90% of cases worldwide, the risk of a first myocardial infarction is related to nine independent, potentially modifiable, risk factors: an abnormal blood lipid profile, smoking, hypertension, diabetes mellitus, abdominal obesity, diet, alcohol, physical activity and psychosocial factors such as depression [2].

The effect of modifying these risk factors is clearer for some behaviours than for others. For example, there is substantial evidence that smoking cessation [3] treatment to reduce blood pressure; treatment to reduce blood lipids and adequately control diabetes [4]; adopting a Mediterranean diet [5, 6]; and increasing levels of physical fitness [7] reduce cardiovascular mortality. A body mass index (BMI) above 25 kg/m^2^ is associated with a 23% increased risk of developing CVD [1]; however, limited success at achieving long-term weight loss, together with repeated attempts to lose weight followed by weight gain, limit the potential benefits of weight loss. It has been suggested that such weight cycling is physically harmful but the evidence surrounding this is conflicting [8–11].

The National Institute for Health and Clinical Excellence recommend that interventions for obese individuals comprise components that address diet and physical activity and incorporate behavioural change techniques. It recommends that individuals reach and maintain a realistic target weight loss of 5–10% of their original weight through a weekly weight loss of 0.5 kg to 1 kg [12]. Although many overweight people are able to lose weight in this way, several meta-analyses of randomised controlled trials (RCTs) show a large proportion of these people are unable to maintain this weight loss [13–15] and may regain more weight than they lose [16].

Lack of a sustained change in weight and, hence, a lack of a reduction in long-term chronic disease risk may be due to an emphasis on food restriction, leading to hunger and feelings of deprivation or preoccupation with food [17–19], which may in turn trigger overconsumption. This overconsumption may lead to weight gain accompanied by feelings of low self-esteem, depression and guilt, which trigger further overconsumption. Further attempts are made to restrict eating and a cycle of dieting and bingeing, weight loss and weight gain are perpetuated, with little long-term gain in CVD risk reduction [20–23].

Interventions have been developed to focus on the health gains of dietary change, physical activity and psychosocial well-being in those who are overweight or obese, rather than on weight loss. RCTs have shown that these ‘health, not weight loss, focused’ (HNWL) programmes may have a greater effect on reducing cardiovascular risk factors, such as improving blood lipid profile and blood pressure and reducing depression, compared with conventional weight loss (CWL) programmes [24, 25]. Two narrative review papers have been published on these RCTs [26, 27], one systematic review containing results from trials with various study designs [28] and one systematic review of RCTs and quasi-controlled trials [29]. These have reported favourable outcomes of HNWL programmes; however, they lack quantifiable pooled estimates of HNWL approaches in comparison to CWL programmes at specific times of follow-up. To date, no meta-analysis has been conducted on RCTs specifically comparing HNWL-focused programmes with CWL programmes on long-term outcomes and, as such, no clinical recommendation for or against the effectiveness of HNWL compared to conventional care can be made. This meta-analysis of RCTs provides quantifiable pooled estimates of HNWL approaches in comparison to CWL programmes on cardiovascular risk outcomes to inform clinical practice and future research.

## Aim

To conduct a systematic review and meta-analysis to compare the effects of HNWL programmes with those of CWL programmes on CVD risk factors in adults with a BMI greater than 25 kg/m^2^.

## Methods

### Study inclusion criteria

Only RCTs or cluster RCTs which compared HNWL programmes with CWL programmes in overweight or obese adults were included.

HNWL programmes were defined as any programme promoting an increase in physical activity and healthy eating without a primary focus on weight loss. The focus was instead to improve physical and mental health through addressing a variety of aspects including lifestyle, emotional, social and spiritual factors. CWL programmes were defined as any diet, exercise or behavioural programme, or a combination of these, focusing primarily on achieving a weight loss target of between 0.5 kg/week and 1 kg/week, with regular weight monitoring and a conscious effort to reduce dietary energy intake.

### Outcome measures

Outcome measures were based on six of the nine main risk factors for myocardial infarction described previously [2]. Trials included at least one of these outcome measures to be eligible. The six risk factors chosen were those most typically measured as part of weight management interventions and excluded the assessment of changes in the prevalence of smoking and diabetes. Alcohol intake was not assessed as no studies reported on this. Included studies had a minimum follow-up period of two months after the end of treatment.

#### Primary outcomes

These were the following physiological markers of cardiovascular risk: blood lipids; blood pressure and body weight. In trials using more than one measure for any outcome, the measure with the strictest criteria was preferred (e.g. measured over self-reported weight).

#### Secondary outcomes

These were those risk factors that mediated the primary outcomes, but that were also known independent cardiovascular risk factors. They included diet, physical activity, and psychosocial well-being, each of which was measured in a variety of ways. Measures of the same dimension were pooled together and included only those studies that measured these outcomes with validated tools.

### Search methods for identification of studies

The following databases were searched: Cochrane Central Register of Controlled Trials 1960 to 3 March 2019, ASSIA (Proquest, 1987 to 5 April 2019), MEDLINE (Ovid, 1946 to 3 August 2015; ebsco, 3 August 2015 to 5 April 2019), PsycINFO (Ovid, 1967 to Aug 2015; ebsco, Aug 2015 to 5 April 2019), CINAHL (Ovid, 1981 to Aug 2015; ebsco, Aug 2015 to 5 April 2019) and EMBASE (Ovid, 1974 to Aug 2015)

The search strategies included MeSH terms and keywords combined with ‘OR’ to for each PICO category of population, intervention, comparator and outcome; the categories were combined with AND. The first search strategy was developed for Medline in Ovid and adapted for use with the other databases using their unique subject heading indexes. Terms or limiters were applied for study type depending on functionality of each database (see Additional file 1 for full search strategies for each database).

Any relevant ongoing or unpublished trials were identified through searching trial registers: ClinicalTrials.gov (https://www.clinicaltrials.gov/); WHO International Clinical Trials Registry Platform Search Portal (http://apps.who.int/trialsearch/); National Institute for Health Research search portal (https://discover.dc.nihr.ac.uk/portal/home) and National Research Register Archive (portal.nihr.ac.uk/Pages/NRRArchive.aspx), accessed via (https://bepartofresearch.nihr.ac.uk/); UK Clinical Trials Gateway - Current Controlled Trials (http://www.isrctn.com/) and University Hospital Medical Information Network Clinical Trials Registry (www.umin.ac.jp/ctr/);

The reference list of included studies, background articles and the narrative review articles described above were hand searched to identify any additional relevant studies.

Commercial and non-profit organisation websites were also searched for additional relevant information: HAES UK (http://www.healthateverysize.org.uk/), Association for Size Diversity and Health (https://www.sizediversityandhealth.org/), National Association to Advance Fat Acceptance (NAAFA) (http://www.naafaonline.com/dev2/) and the resource list for the HAES curriculum (http://haescurriculum.files.wordpress.com/2013/07/haes-curriculum-resource-list.pdf). The authors of relevant papers where trials were ongoing or data were missing were contacted.

### Selection of studies

NK and DL checked the titles and abstracts of studies generated by the search. Full-text copies of papers reporting selected trials were obtained. NK and DL independently reviewed the trials, either accepting or rejecting them in accordance with the eligibility criteria. Any disagreement regarding study inclusion was resolved through discussion with a third author (GF). Where a number of reports of the same study were come across, the reports were allocated to a single study ID and the data was used only once.

### Data extraction and management

For each trial, NK extracted the data and one other author checked data extraction. A data collection form was used to record details of the study methods, participant characteristics and outcomes.

### Assessment of risk of bias in included studies

Risk of bias was assessed using the guidelines from the Cochrane Handbook for Systematic Reviews of Interventions [30]. Random sequence generation, allocation concealment and incomplete outcome data were graded as having a high, low or unclear risk of bias by NK, and this grading was checked by one other author for each study. The ‘risk of bias’ judgements were summarised across different studies for use in sensitivity analysis.

### Measures of treatment effect

Many of the outcomes (e.g. blood lipids, blood pressure, weight) were continuous data and analysed as mean differences with 95% confidence intervals, in order to compare the change in outcome between the intervention and control arms. Data presented as a scale was entered with a consistent direction of effect. Some of the outcomes used ordinal scales (e.g. measures of psychosocial well-being, levels of physical activity). Where possible (i.e. with scales of five or more ordinal categories), these were treated as continuous data. If there was variation in the scales used to measure the same outcome, these outcomes were analysed using standardised mean differences and 95% confidence intervals (e.g. for body dissatisfaction and self-esteem).

Where a study did not report standard deviation (SD), it was calculated from the standard error of the mean (SEM) information provided in the studies. SD can be obtained from the SEM by multiplying by the square root of the sample size: SD = SEM × √*n*. The SEMs were also used to calculate the SD for mean differences using formulas.

### Dealing with missing data

Investigators or study sponsors were contacted to verify key study characteristics and obtain missing numerical outcome data where possible. Where this was not possible and the missing data were thought to introduce serious bias, the impact of including such studies on interpretation of the results using sensitivity analyses was explored.

### Assessment of heterogeneity

Inconsistencies across study results were identified and analysed using forest plots. The overlapping confidence intervals were observed and the *I*^2^ statistic was used to measure heterogeneity among trials in each analysis. Significant heterogeneity was defined as a *P* value of less than 0.05 for the Chi^2^ statistic (*Q*). Heterogeneity was described as a percentage using the *I*^2^ statistic (*I*^2^ = [(*Q* − degrees of freedom)/*Q*] × 100%). Where there was significant heterogeneity, the pooled estimate for this analysis was provided and heterogeneity was investigated as described below. If *I*^2^ remained over 75%, use of a random-effects model was planned but this was unnecessary as sub-analysis dealt with heterogeneity adequately.

#### Subgroup analysis

The Mantel-Haneszel fixed-effect model was used for pooling results; where significant heterogeneity was found, sub-group analysis was carried out. Subgroup analyses were used to take into account different lengths of follow-up and enhanced programmes. Enhanced interventions potentially included additional elements that might influence any findings (e.g. one HNWL programme promoted calorie restriction for the first 2 weeks).

#### Sensitivity analysis

Sensitivity analyses were conducted on studies with a high risk of bias due to greater attrition rates and omission of information relating to intention to treat (ITT) analysis. These allowed us to identify and review the influence of the studies with a high risk of bias.

### Assessment of reporting biases

Assessment of the risk of selective outcome reporting was planned across the studies using a funnel plot, if sufficient studies were available. However, with fewer than 10 articles in the review, Sterne et al. [31] advise that a funnel plot for an asymmetry analysis should not be used. This is because the outcomes are usually too low to distinguish chance from genuine asymmetry.

## Results

### Search results

In total, 20,242 potentially relevant studies were identified from searches of bibliographic databases. A further eight studies were found from websites relating to the HNWL programme. Another 31 studies were selected from the reference lists of studies that we had already identified as relevant. Of the total 20,242 studies, 3,026 studies were duplicated and removed.

Of the total 17,216 remaining studies, 17,106 studies were considered irrelevant and excluded by title and abstract. Our screening resulted in 110 papers with a Cohen’s kappa of 0.74. Two authors independently assessed the 110 studies according to the eligibility criteria. Ninety-eight articles were excluded at this stage. In 10 articles, the intervention did not have a control group and in 15 the control was not CWL. In 49 articles, the intervention did not follow the HNWL philosophy because it promoted an energy-restricted diet. Twelve studies were excluded because there was no randomisation reported; nine studies were excluded because participants with a BMI under 25 kg/m^2^ were not excluded, two studies excluded because they were protocols in their recruitment phases and one study was excluded because the duration was less than 2 months. Finally, nine articles were excluded because they were duplicates. Our full-text review resulted in 12 articles identified with a kappa of 0.87. We addressed any disagreements directly through discussion involving the two raters and settled these by collaborative review.

**Fig. 1:**
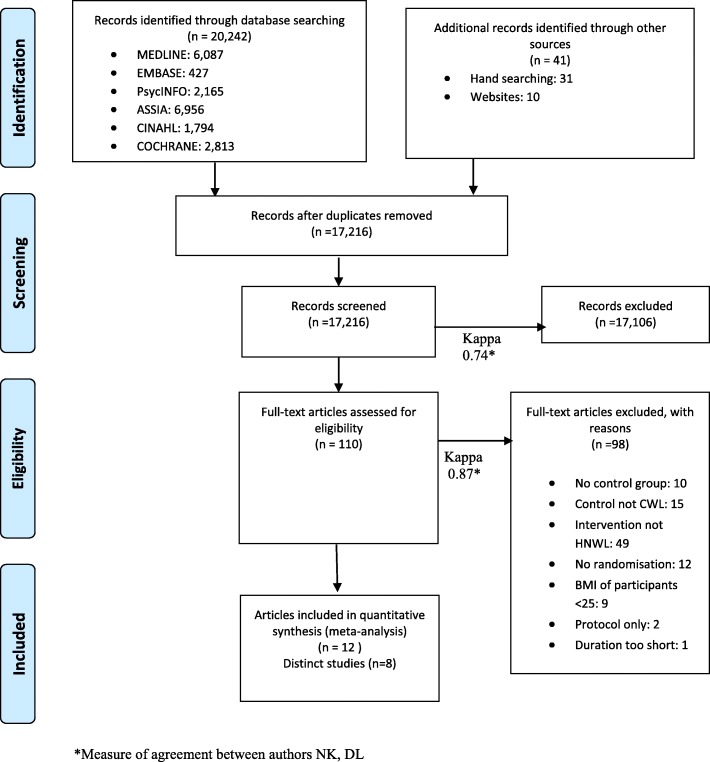
PRISMA flowchart

Of these twelve articles, some articles reported on the same studies (Table 1). Therefore, eight distinct studies were included in the final review (Fig. 1).

**Table 1:** Characteristics of included studies

Included studies	Location	Age (years) Mean (SD)	BMI (kg/m^2^) Mean (SD)	Men (*n*)	Women (*n*)	Methods	Participants	Intervention	Attrition
Ash et al., 2006 [32]	Brisbane, Australia	48 (13)	34 (5.5)	47	129	Randomisation: number table. Allocation concealment unclear. Weekly follow- up for 8 weeks, monthly for 6 months, final 12 month follow-up.	Inclusions: BMI > 27 Exclusions: Co-morbidities, non-English speakers, Cognitively impaired.	HNWL (Fat Booters Incorporated (FBI)): *n* = 57 CWL (individualised Dietetic Treatment (IDT)) : *n* = 65 Control group (Information Booklet only (BO): *n* = 54 Delivered by dietitians and nutrition experts	ITT analysis use of generalised estimating equations. Attrition(at 12 months): FBI: 54% CWL : 32% BO: 63%
Bacon et al., 2005 (Bacon et al., 2002)[25]	California, USA	40.7	36.3	0	78	Randomisation: stratified by BMI, eating behaviour and physical activity level. Allocation concealment not recorded. 12, 24, 42 and 104 weeks follow-up.	Inclusions: Dietary restraint>15 BMI > 30 Age 35-40 Exclusions: Co-morbidities Smokers Not Caucasian	HNWL (HAES): *n* = 39 Delivered by counsellors and those with doctorates CWL (LEARN): *n* = 39 Delivered by dietitians	No ITT analysis Attrition (at 24 months): HAES: 8% LEARN: 42%
Crerand et al., 2007 [33] (Wadden et al., 2004) [34]	Philadelphia, USA,	44.2	35.9	0	123	Randomisation and allocation concealment unclear. Weekly group session for 20 weeks, biweekly weeks 20-40. Week 65 follow-up	Inclusion: BMI 30-43 kg/m2 Exclusion: Co-morbidities lost > 5 kg or used weight loss medications in past 6 months	HNWL (non-dieting approach (ND)): *n* = 39 CWL (balanced-deficit diet (BDD)) : *n* = 43 MR (meal replacement plan): *n* = 41 Delivered by qualified clinical psychologist and registered dietitian	ITT analysis, last observation carried forward with assumed weight gain and sensitivity analysis Attrition (at week 65): ND: 74% BDD: 60% MR: 68%
(Keller, 1999) [35] Goodrick et al, 1998 [36]		40	33	0	219	Randomisation and allocation concealment unclear. 24 weeks of weekly treatment followed by 26 biweekly meetings for 12 months	Inclusion: Female Age 25–50 14 to 41kgs overweight Exclusions: Registered with a weight loss programme Co-morbidities Smoker	HNWL (Non-diet treatment (NDT)): *n* = 78 CWL (dieting treatment (DT)): *n* = 79 Waitlist control (WLC): *n* = 62 Delivered by instructors, a registered dietitian and a qualified psychotherapist specialised in eating disorders.	ITT analysis was carried out with baseline values carried forward and sensitivity analysis Attrition (at 18 months): NDT: 21% DT: 18% WLC: 6 % (6 months)
Mensinger et al., 2009 [37] (Mensinger, Calogero, and Tylka, 2016) [38]	Pennsylvania, USA	39.6	38	0	80	Computer generated randomisation. Allocation concealment using sealed opaque envelopes labelled with the sequential randomisation numbers. 6 and 24 month follow-up	Inclusion: Women aged 30–45 BMI 30–45 Physically inactive Pre-menopausal Exclusion: current smokers, did not speak fluent English Co-morbidities	HNWL (HUGS) *n* = 40 CWL (LEARN) *n* = 40 Delivered by trained group facilitator	ITT with SPSS MIXED and restricted maximum likelihood Attrition (24 months): (HUGS): 53% (LEARN): 48%
Rapoport, Clark and Wardle, 2000 [23]	London, UK	47.5	35.3	0	84	Randomisation and allocation concealment unclear. 10 sessions. 6 and 12 month follow-up.	Inclusion: age18–65 BMI > 28 approved by their GP for treatment Exclusion: involved with any other weight management programme Co-morbidities	HNWL (Modified cognitive-behavioural treatment) *n* = 37 CWL (cognitive behavioural treatment) *n* = 38 Delivered by Clinical psychologist, exercise specialist, dietitian, health psychologist trained in CBT methods.	No ITT analysis was reported Attrition (at 12 months f-up): Modified cognitive-behavioural treatment: 16% Cognitive behavioural treatment: 16%
Sbrocco et al., 1999 [39]	Maryland, USA	41.3	32.6	0	24	Randomisation and allocation concealment unclear. 13 weekly sessions post treatment 3, 6 and 12 months follow up	Inclusion: Healthy (GP verified) Exclusion: Lost >4.5 kg in previous month or > 9 kg previous 6 month Smoker	HNWL (behavioural choice treatment (BCT)): *n* = 12 CWL (Traditional Behaviour Treatment (TBT)) : *n* = 12 Delivered by : Clinical social worker/psychologist, or a psychology graduate	No ITT analysis Attrition(at 12 months f-up): BCT: 8% TBT: 0%
Tanco, Linden and Earle, 1999 [40]	Vancouver, Canada, British Columbia	Age > 19	39.6	0	62	Randomisation and allocation concealment unclear 12 weeks treatment with 6 months follow up	Inclusion: women > 19 BMI > 30 kg/ m^2^ 3 weight cycles over at least 10 years Exclusion: Co-morbidity which would disallow increased physical activity.	HNWL (Cognitive treatment (CT)): *n* = 21 CWL (Standard behavioural weight management program (BT)): *n* = 21 Wait-list control group: *n* = 20 Delivered by psychology graduates	No ITT analysis Attrition (at 6 month f-up): CT: 57% BT: 43% Wait-list control group: 60%

### Characteristics of included studies

#### Participants, sample sizes and settings

Participant numbers in the eight studies ranged from 24 [39] to 219 [36]. A total of 846 participants were included in our review, 799 women and 47 men. In seven of the eight studies, the participants were exclusively women [23, 25, 33, 38–40, 41] and the remaining study [32] recruited men and women. The high proportion of women-only studies may be because the HNWL ethos has been borne out of a feminist philosophy and targets women in particular [41]. The mean age of participants across studies was 43 ± 8.92 years, although one study failed to provide age data [40]. The mean body mass index (BMI) across study participants was 35 ± 5.4 kg/m^2^. Five of the studies were conducted in the USA [25, 33, 36, 37, 39], one in Australia [32], one in the UK [23] and one in Canada [40].

The trials were undertaken in a range of outpatient settings, including general practitioner’s (GP) surgeries [23], a hospital [32], community centres and health centres [23]. The trials were advertised locally.

Interventions were delivered by qualified individuals including registered dietitians [23, 25, 32, 33, 36], trained facilitators [37], counsellors with doctorates in nutritional physiology [25], psychotherapists with a background in eating disorders [36], psychologists [23, 33, 39, 40], a clinical social worker [39]. These individuals were also responsible for gathering participant data, providing group support, dietetic assistance and follow-up sessions across the eight studies.

#### Interventions

Included studies focused on two comparison groups (HNWL and CWL). Four of the studies also incorporated a third group; two were waitlist controls [36, 40], one was a ‘no intervention’ control group [32] and one was a meal replacement group [33]. The characteristics of participants were checked across all groups to verify randomisation, but only extracted and used data from the HNWL and CWL programmes.

##### HNWL programmes

Duration

The length of the sessions ranged from 1 [36] to 2 h [23, 40]. Weekly sessions continued for 8 weeks in some programmes [32] and up to 78 weeks in others. Follow up was 24 weeks in some studies [40] and as much as 104 weeks in others [25, 37]. We addressed the differences in outcomes this would have led to, by reporting the results as four phases of follow-up: the period between 8 and 12 weeks, from 20 to 26 weeks, from 40 to 52 weeks and the period between 65 and 104 weeks.

Aims

The primary aims of the intervention groups included improved health and well-being [25, 36, 37] and positive lifestyle change [32]. Participants in Rapoport, Clark and Wardle’s [23] study had a primary aim of weight management, rather than weight loss, through permanent lifestyle change, no goals to restrict energy intake were set.

Approach to weight and eating

The dietary changes recommended in Rapoport, Clark and Wardle’s [23] study were based on the Health Education Authority’s (HEA) ‘Balance of Good Health’ plate model. Participants were not given precise goals relating to energy intake, and they were permitted to eat foods that they previously avoided in reasonable amounts.

Participants were advised to expect a slower reduction of weight over a longer timescale, than in the CWL programmes, in order to achieve a long-lasting result. In Crerand et al.’s [33] study, a group of registered dietitians gave six lectures on the subject of healthy eating whilst avoiding prescribed restrictions on energy intake. At week six, the participants in the study were encouraged to adopt a new eating plan without dieting. The plan included several instructions: participants would eat at least every 4 h in order to avoid physical hunger pangs, they would consume foods that they enjoyed without restrictions, they would select foods based on their nutritional value, and they would not monitor their weight throughout the process.

Participants were encouraged to respond to internal cues of physical hunger and satisfaction. They were taught principles of intuitive eating in order to reject the diet mentality, recognise their hunger and respect when they felt full [23, 25, 37, 39, 40]. All studies addressed issues of body acceptance, eating behaviour avoiding diet cycles, healthy eating, enjoyable physical activity, rejecting social pressure and accessing social support. In doing so, they aimed to improve self-awareness, self-confidence and self-esteem [25, 32, 36, 37, 39].

Participants were asked not to specifically reduce their calorie consumption or monitor their weight [25, 33, 39, 40].

Physical activity

All the HNWL groups were advised to undertake an enjoyable type of physical activity with no specific exercise regimen enforced. Exercise recommendations were generally less formal in the HNWL programme than the CWL programmes, although in some studies the programmes followed a similar pattern of exercise in both the HNWL and CWL groups [36, 39]. In Rapoport, Clark and Wardle [23], exercise routines were designed and tailored to the needs of study participants.

Behaviour change techniques

Ash et al.’s [32] study group focused on self-improvement, self-sufficiency and prevention of relapse through the application of cognitive therapy. It followed a patient-centred approach that promoted and included a booklet centred on cognitive behaviour therapy principles relating to nutrition. Bacon et al. [25] encouraged acceptance of body shape and focused on how to live a fulfilling life at any weight. Crerand et al. [33] educated participants on obesity, its causes and effects on self-esteem, body image and quality of life. Participants were given selections from *Self-Esteem Comes in All Sizes,* a book containing methods to increase self-esteem, body image and life satisfaction. The first stage of the intervention in Goodrick et al.’s [36] study entailed a psychotherapeutic treatment based on the modern ideal of female body shape. It focused on identifying problems with self-esteem and body image before promoting healthy eating habits and exercise. Rapoport, Clark and Wardle [23] employed behavioural and cognitive techniques with elements of other approaches, including psycho-educational methods and feminist discourse. Rapoport, Clark and Wardle [23] promoted lifestyle changes through self-awareness and autonomy, asking participants to keep journals throughout the process. Sbrocco et al.’s [39] approach was based on Behavioural Choice Treatment (BCT), discussing how food choice, exercise and eating behaviour play a crucial role in suppressing hunger, losing weight and improving health. General rather than individual support was given. Tanco, Linden and Earle’s [40] study considered all ancillary issues that might contribute to weight gain, e.g. sexual abuse. Through discussion, participants considered the role of personal experiences in weight management. Social pressures were also discussed and participants were asked to engage in sessions by sharing personal views, feelings and experiences.

Each of the following behaviour change techniques [42] were used in all nine of the HNWL interventions: goals and planning (number 1), feedback and monitoring (number 2), social support (number 3), shaping knowledge (number 4), natural consequences (number 5), repetition and substitution (number 8), identity (number 13) and self-belief (number 15).

##### CWL programmes

Duration

As with the HNWL interventions, most participants in the CWL programmes attended a weekly group session [25, 33, 36–40] that ranged from 1 [36] to 2 h in duration [23, 40]. The durations of the CWL programmes were matched to the corresponding HNWL programmes.

Aims

Despite minor variations in their approaches, all groups followed the CWL philosophy in practice with the primary objective to achieve weight loss. Participants assigned to the CWL group were given an initial nutritional assessment and given a bespoke diet plan that aimed to achieve weight loss of 0.5–1 kg a week. The CWL programmes aimed to achieve this through self-control, physical activity and weight monitoring.

Approach to weight and eating

Several CWL groups followed the LEARN programme, which promotes dieting as fundamental to weight control [25, 33, 36, 37]. The programme’s primary goal is weight loss through gradual and sustainable lifestyle changes based on increased physical activity and decreased energy intake [23]. To achieve this, LEARN promotes positive eating behaviour, good nutrition, social support, exercise, decreased fat intake, monitoring of weight and addressing self-esteem and its relationship to weight loss and weight gain. It relies upon methods of self-monitoring, stimulus control, social support, problem-solving, goal setting and relapse prevention [25, 33, 36, 37].

Nutritional education was promoted through methods of reducing the intake of fatty foods, increasing the consumption of complex carbohydrates and eating a wide variety of foods [23, 25, 33, 36, 37]. Participants were taught to restrict their fat intake to 40 g a day and to keep food diaries [39]. Bacon et al. [25] instructed individuals to follow a diet where 30% of their daily energy intake came from fats, 15% from proteins and the remaining energy from carbohydrates. In Crerand et al. [33], relapses were avoided through monitoring food consumption and increasing physical activity levels, undertaking a paced eating speed and using social support systems. Through these tools, participants learned to restrict their energy and fat consumption, keep food diaries and monitor their weight. The CWL group in Rapoport, Clark and Wardle’s [23] study were encouraged to make healthy eating choices and select food options that were low in fat, low in sugar, high in fibre and low in salt. Further to this, they were provided with sample menus, strategies for regularising eating habits, managing overeating, modifying recipes, reading food labels, coping with eating out and strategies for maintaining weight loss.

The key difference between HNWL and CWL was that CWL prescribed a specific daily energy restriction and meal plans to follow and promoted weight monitoring. All of the CWL groups followed a weight loss plan to achieve weight loss of at least 0.5–1 kg per week. This was achieved by restricting calorie intake to 1,200 kcal/day [23, 39] or to 1200–1,500 kcal a day [40]. Exact kilocalories per day prescription was not specified in Ash et al. [32], although the programme was designed to achieve weight loss of 0.5–1 kg/week. The remaining studies did not report the exact amount of energy restriction imposed on participants [25, 33, 36, 37], but as reported by Crerand et al. [33], all of these studies followed the LEARN programme and therefore restricted calorie intake by a self-imposed diet within a range between 1,200 and 1,500 kcal a day.

Behaviour change techniques

Crerand et al.’s [33] group was educated on the relationship between self-esteem and weight and discussed the emphasis in the media on thinness. Rapoport, Clark and Wardle [23] used cognitive and behavioural approaches to teach healthy weight loss. The topics in these sessions included self-monitoring, identifying personal triggers, using social support systems, focusing on goals and positive reinforcement.

Sbrocco et al. [39] asked participants to pinpoint the true causes behind overeating and to identify alternative methods of coping with stress. Weight management was approached through behavioural techniques. Tanco, Linden and Earle [40] used psycho-educational methods focused on the specific effects of weight loss and gain. These studies used Behaviour change techniques (BCT) from the BCT taxonomy [42]**.** with respect to goals and planning (number 1), feedback and monitoring (number 2), social support (number 3), shaping knowledge (number 4), natural consequences (number 5), repetition and substitution (number 8), rewards and threats (number 10) and self-belief (number 15). Many of these BCTs were features of both the HNWL and CWL programmes, although number 13 (identity) and number 10 (rewards and threats) were solely features of the HNWL [40] and CWL [23] programmes, respectively.

Physical Activity

All of the CWL groups across the nine included studies encouraged their participants to increase their level of physical activity. Participants in Rapoport, Clark and Wardle [23] were advised to exercise for 30 min, three times per week. In Crerand et al. [33], physical activity included walking, or other aerobic activity, for 150 min per week. At the end of week 20, this was increased to 180 minutes per week. Participants in Sbrocco et al. [39] and Tanco, Linden and Earle’s [40] studies exercised five times a week during the treatment and reduced this frequency to three times a week over the follow-up period. No formal exercise groups or routines were offered and participants kept track of their physical activity via a daily exercise log [39].

#### Enhanced programmes

There were several studies where either the HNWL programme or the CWL programme contained enhanced elements that could potentially result in greater differences in weight and heterogeneity when pooling the results. In Crerand et al. [33], the HNWL participants received an intervention in three phases. The first phase was implemented across the first six weeks and focused on weaning subjects from their existing diets; the second phase, occurring between week six and week 20, had subjects adopt a specific eating plan with a goal of eating at least every 4 h to prevent hunger and where no food groups were restricted; the final phase focused on improvement of body image and self-esteem through therapy sessions held between weeks 20 and 40.

In Sbrocco et al. [39], the HNWL participants were prescribed 1800 kcal/day (7534 kJ) for the first 2 weeks of the study. Therefore, initially, HNWL had an energy restriction which was likely to lead to significant weight loss in this group.

As described above, although these programmes were enhanced in HNWL, beyond the standard interventions, each was enhanced in different ways and it was not appropriate to combine them.

In another study, there was an enhanced aspect to the CWL intervention. After 8 weeks of treatment, participants in the HNWL programme demonstrated marked improvement in their psychological well-being, and individuals in the CWL were provided with a further 4 weeks of treatment as a consequence. They studied the principles taught in the HNWL sessions. In order to ensure both groups received the same contact, the participants in the HNWL programme were also offered 4 further weeks of sessions where they reviewed the topics that were previously covered in the HNWL condition [40]. This may have potentially led to a smaller difference between the intervention and control groups, particularly in respect to outcome measure of psychological well-being, so this needs to be born in mind in interpreting the results.

#### Risk of bias in included studies

**Fig. 2:**
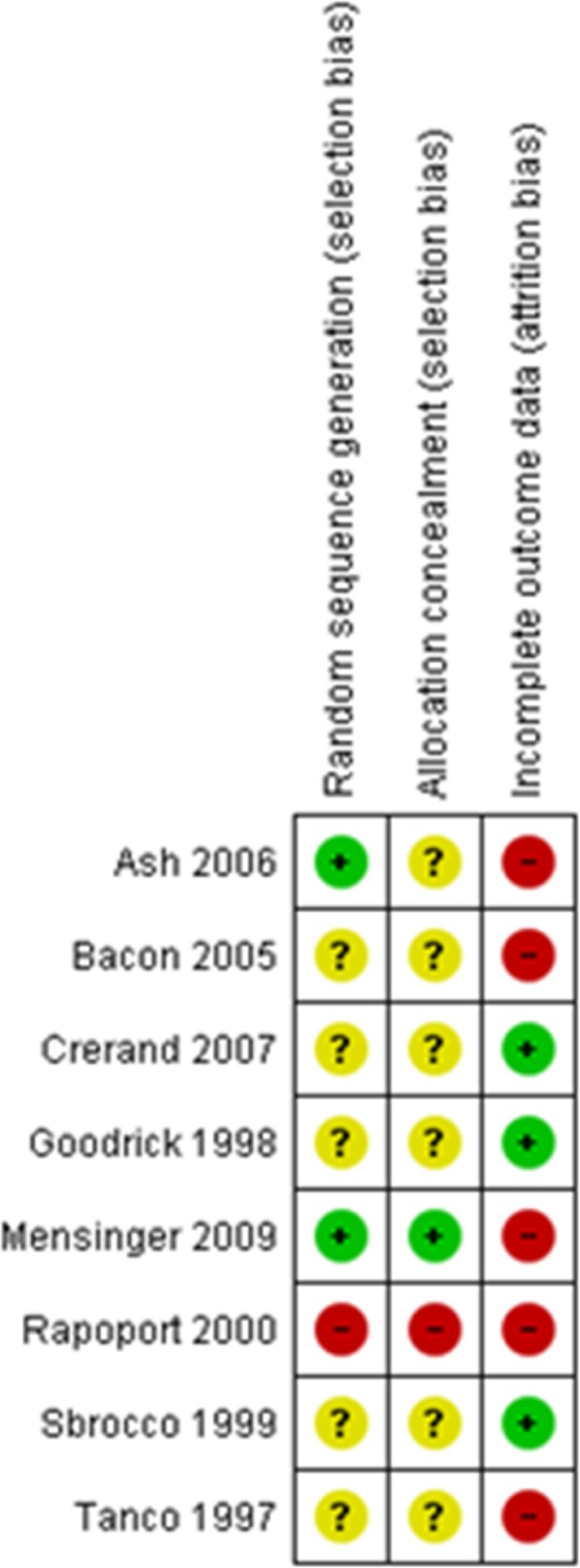
Risk of bias summary: results of the assessment of each risk of bias item for each included study

Information concerning risk of bias for each study can be found in Fig. 2, and summarised below. This includes information on the likelihood of selection bias (including randomisation and allocation concealment) and attrition bias. Each study is classified on each bias as presenting a low risk, a high risk or an unclear risk of bias.

##### Allocation (selection bias)

A large section of studies neglected to report the method used to generate random allocation sequences or information concerning allocation concealment—neither of these were reported in five of the eight studies [25, 33, 36, 39, 40]. Two studies used methods of randomisation with a low risk of bias. Ash et al. [32] used a table populated with random numbers to generate the randomisation sequence. Mensinger et al. [37, 38] used a computer-generated randomisation programme that was devised by a statistician to assign interventions to participants. One study [23] was found to have a less robust method of randomisation that forced alternate allocation of interventions: in the first cohort of participants, treatments were allocated by the toss of a coin. In subsequent cohorts, allocation was alternated to ensure that every form of treatment was represented at specified times of the day.

The method of allocation concealment was only reported in one study Mensinger et al. [37, 38] and was found to have a low risk of bias. Mensinger et al. [37, 38] instructed an assistant to place folded index cards containing group assignment information into sealed, opaque envelopes. These were labelled with the sequential numbers from the randomisation scheme before they were given to participants in the study.

##### Incomplete outcome data (attrition bias)

There were concerns relating to attrition bias across all of the studies. Most studies were found to present a high risk of bias due to high attrition rates (up to 74%). Intention to treat (ITT) analysis was carried out in some studies using modelling techniques or data carried forward with sensitivity analysis, such that ITT analyses were compared to the results from associated completer case analyses and similar results were found [36]. In Sbrocco et al. [39] a low attrition rate (i.e. less than 15%) was reported.

### Data synthesis

Findings were stratified according to the length of the follow-up period. The results pooled revealed high levels of heterogeneity because the studies had varied lengths of follow-up treatment stages. This was addressed by reporting the results as four phases of follow-up treatment based on the timing of data collection in the included studies. These became Period 1, 8-19 weeks; Period 2, 20-39 weeks; Period 3, 40-51 weeks; and Period 4, 52-104 weeks.

All data reported in all studies was captured by these time periods and no data was missed out.

### Effects of interventions

In this report, the meaning of being ‘in favour of’ is that the change reported is clinically better in the ‘favoured’ programme than in the comparator. Results of all outcomes and presentation of meta-analysis for all data is available as supplementary material online (Additional file 2). For reasons of space, within this paper, only the long-term effects of the more commonly used clinical measures are presented here.

### Primary outcomes

Each of the eight studies provided data on weight. Only three studies, reported in four papers [23, 25, 37, 38], reported data on blood lipids and blood pressure.

#### Total cholesterol-HDL ratio

**Fig. 3:**
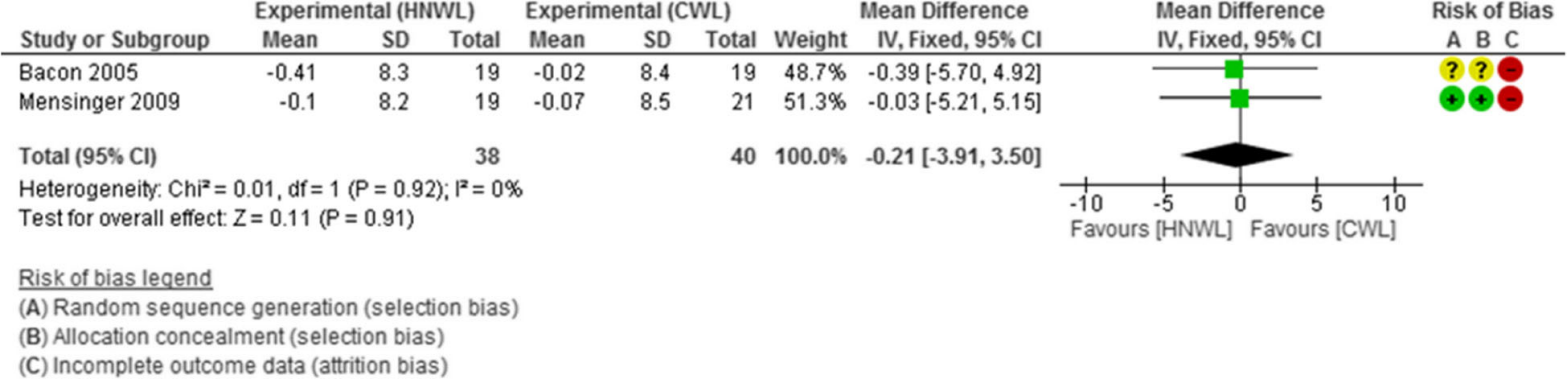
Meta-analysis of total cholesterol-HDL at 53–104 weeks

The total cholesterol-HDL ratio increased in participants in both intervention groups. However, the increase was less in the CWL programmes. The mean difference [95% CI] in the change in total cholesterol-HDL ratio was slightly in favour of the CWL programme by magnitude of (− 0.16, [− 4.51, 4.18]) at weeks 8 to 19, − 0.28 [− 3.68, 3.12] at weeks 20 to 39, and − 0.12 [− 4.30, 4.06] at weeks 40 to 52 (see Additional file 2). It slightly favoured the HNWL programmes during the longer follow-up period between weeks 53 and104 (− 0.21 [− 3.91, 3.50]). This data was based on just 2 low-moderate quality studies (Fig. 3).

#### Blood pressure

**Fig. 4:**
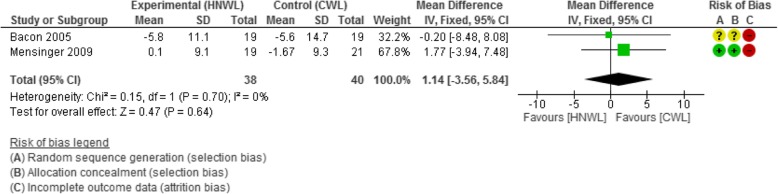
Meta-analysis systolic BP change at 53–104 weeks

**Fig. 5:**
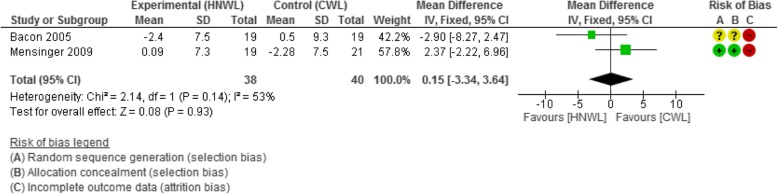
Meta-analysis of diastolic BP change at 53–104 weeks

The mean difference in change in systolic and diastolic blood pressure [95% CI] across participants was in favour of the CWL programmes compared to those on the HNWL programmes at all stages of the follow-up. Those in the CWL programmes had between a 1–3 mmHg and 0–1 mmHg lower systolic and diastolic blood pressure respectively, than those in the HNWL programmes at all time points. This data was based on just 2 low-moderate quality studies, and confidence intervals were wide (Figs. 4 and 5). The meta-analysis for diastolic blood pressure contained heterogeneity of 53% (*p* = 0.14) (Fig. 5), but this was considered moderate and below our a priori protocol for random effects modelling.

#### Weight

The weight loss data was analysed in three ways: firstly, weight loss results from all studies pooled, secondly, a sensitivity analysis excluding data from groups using enhanced programmes, and thirdly, a sensitivity analysis excluding studies found to have a high risk of bias.

##### All studies

**Fig. 6:**
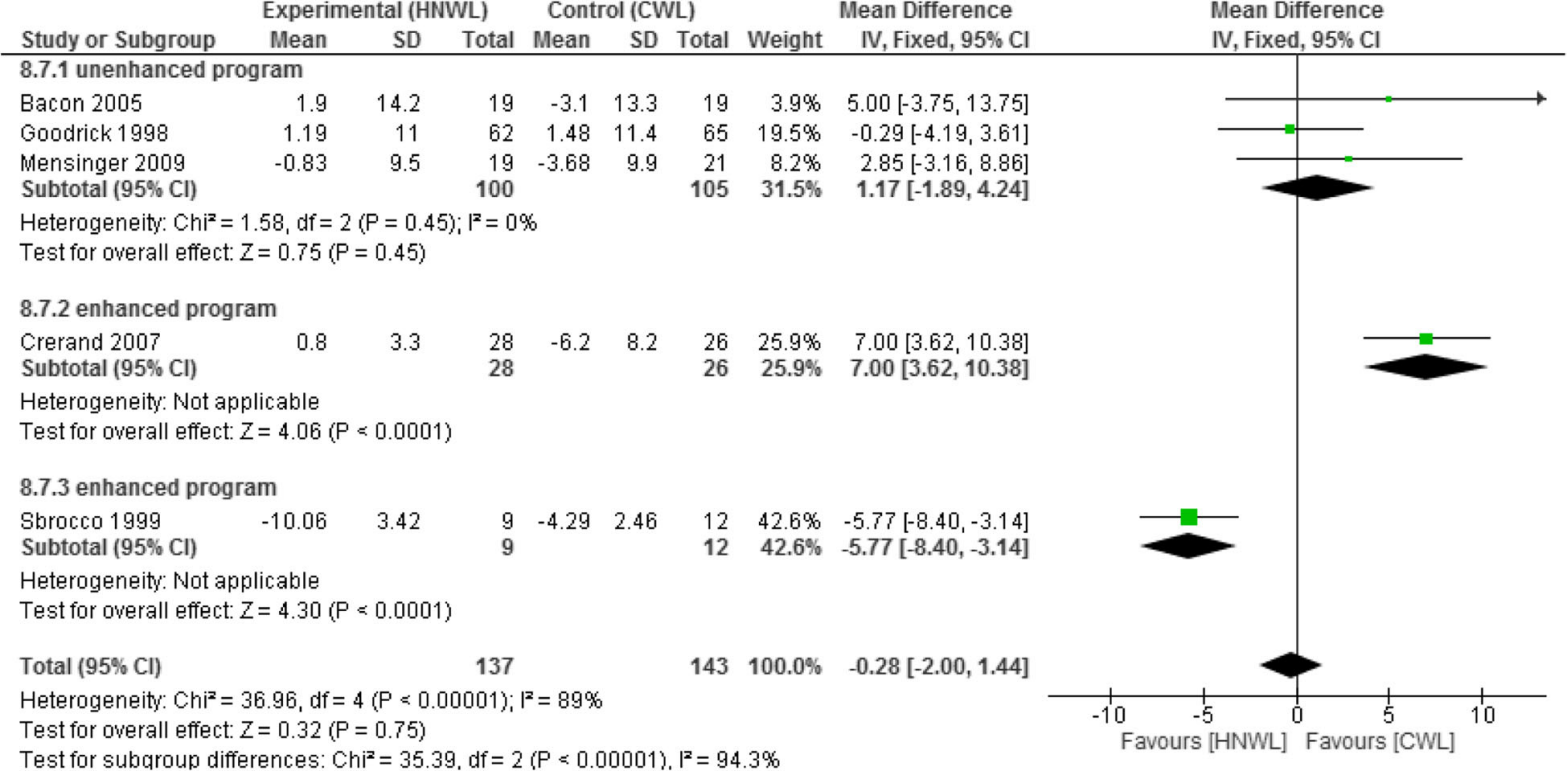
Meta-analysis of weight change at 53–104 weeks

The mean weight loss [95% CI] across participants was greater in the CWL programmes compared to the HNWL programmes, at the end of treatment (− 1.43 kg, 95% CI [− 2.48 to − 0.38] at 8 to 19 weeks), and during the follow-up periods weeks 20 to 39 (− 2.89 kg, [− 4.05 to − 1.72] and 40 to 52 (− 0.05 kg, [− 1.38 to 1.27] (see Additional file 2). At the latest follow-up (weeks 53 to 104), improvements in weight were in favour of the HNWL programmes (− 0.28 kg, [− 2.00 to 1.44]) this contained significant heterogeneity (*I*^2^ = 89%) which was dealt with by firstly a sub-analysis excluding data from groups using enhanced programmes (Fig. 6).

##### Excluding enhanced programmes

Considering only the unenhanced programmes at weeks 8 to 19 there was a greater mean weight loss in the CWL programmes compared to the HNWL programmes (3.09 kg, [1.03, 5.15]. Results were similar for the 20–39 week follow-up period (− 0.69 kg, [− 2.10, 0.72] (see Additional file 2), and at 53 to 104 weeks (− 1.17 kg, [− 4.24, 1.89] (Fig. 6)). The mean weight loss between weeks 40-52 was slightly in favour of the HNWL programme (− 0.16 kg, [− 2.30, 1.99].

##### Excluding studies with high risk of bias

**Fig. 7:**
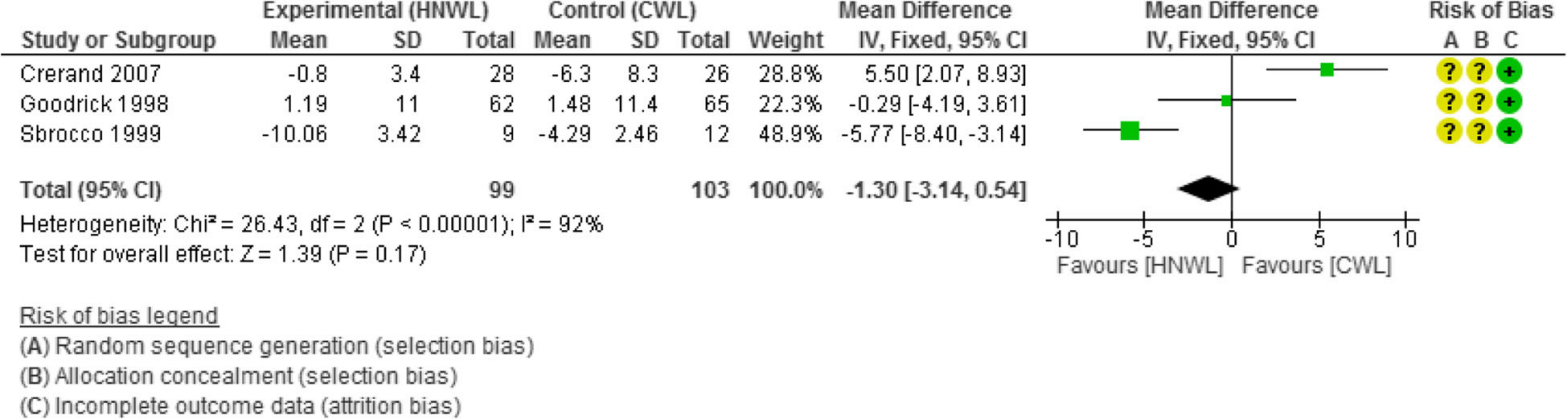
Meta-analysis of the results weight at 53–104 weeks (excluding studies with high bias)

Excluding studies with a high risk of bias at 8–19 weeks and 20–39, the mean weight loss was greater in the CWL than in the HNWL programmes (− 3.09 kg, [− 5.15, − 1.03] and − 6.21 kg [4.42, − 8], respectively). By 40–52 week follow-up, this difference was smaller (− 0.18 kg, [− 1.86, 1.50] (see Additional file 2). By the 53–104 week follow-up, the mean weight loss favoured the HNWL programmes by − 1.3 kg [− 3.14, 0.54] (Fig. 7). However, this data included two enhanced studies so there was high heterogeneity (*I*^2^ = 92). This was dealt with by separating out the enhanced programmes, as per previous sub-analysis, this left us depending on the study of one unenhanced programme [36] (Fig. 7) which showed at 53–104 week follow-up a − 0.29 kg [− 4.19, 3.61] in favour of HNWL.

### Secondary outcomes

#### Dietary intake, physical activity and alcohol intake

Data were available on physical activity levels [23, 25, 32, 36, 38, 39], fruit and vegetable intake, intuitive eating behaviour, [37] eating patterns and nutrient intake [23, 39].

#### Dietary intake

Mensinger et al. [37, 38] reported an increase in participants’ intake of fruit and vegetables. The mean increase was 0.32 portions higher in the HNWL programme (mean (sd); 0.98 (0.82)) compared to those on the CWL programme (0.66 (0.84)). Energy intake in Rapoport, Clark and Wardle’s [23] study showed a reduction over time in both programmes, with very little difference between them at 52 weeks (− 466Kcal/d (HNWL) and − 462 kcal/d (CWL)). There was a greater reduction in percentage energy from fat (5.2 g vs 3.4 g) and increase in the percentage energy from protein (2.2% vs 1.8%) and carbohydrate (3.4 g vs 1.4 g) in the CWL, compared to the HNWL, programmes. The amount of fibre consumed increased in the CWL programmes (1.3 g) and was reduced in HNWL (− 2.7 g). The intake of sucrose was reduced in both programmes, although the HNWL group saw a greater reduction (− 14.3 g vs − 12.6 g).

#### Physical activity

Energy expenditure levels were measured, and this was done in three different ways: Bacon et al. [24] reported daily energy expenditure (kcal/day) and reported exercise in kcal/kg per day, and Rapoport, Clark and Wardle [23] reported MET (metabolic equivalents) hours per week. All of these results were converted to daily energy expenditure in kilocalories per day. Participants on the HNWL programmes had greater energy expenditure at all stages compared to the CWL programmes (MD − 81 kcal/day 95%CI [− 173, 336] 8–19 weeks; − 94 kcal/day [− 17, 171] 20–39 weeks; − 224 kcal/day [− 20, 469] 40–52 weeks; − 9.00 kcal/day [− 80, 98] 53–104 weeks although this latter time relied on one study’s data).

Four further studies reported physical activity [37], regular exercising [40], frequency and duration of exercise sessions across the treatment and follow-up period [39], and the ratio of physical activity in comparison to baseline [32]. The results of Ash et al.’s [32] study showed some differences between the number of participants who were physically active. The likelihood of the participants remaining active in the CWL group compared to the HNWL was decreased at 3 months, but there was no difference at the 6 and 12-month periods. Sbrocco et al. [39] reported an increase of physical activity in the treatment stage of both programmes, but the CWL programme showed a greater change. This continued throughout all follow-up stages after treatment in either programme. In the CWL programme, subjects were more likely to have reduced their physical activity during the longer follow-up period. Tanco, Linden and Earle’s [40] study showed that following treatment, there were more regular exercisers in the HNWL programme than in the CWL programme. However, the proportion did not differ greatly between the two treatment groups. The proportion of participants who exercised regularly in the HNWL programme increased over the course of treatment but this was less so in the CWL programme. In fact, during the same period, there was a decrease in the proportion of individuals in the CWL programme who reported exercising regularly. Mensinger et al. [37, 38] reported an increase of physical activity in the HNWL programme only.

#### Psychosocial wellbeing

Data were available on restrained eating behaviour [23, 25, 33, 39] and pathological eating behaviours’ including binge eating and loss of control [23, 33, 36]. Data were also available on self-efficacy, self-esteem [23, 25, 32, 33, 37, 39], body dissatisfaction [23, 25, 39, 40] and body image [23, 25, 33], depression [23, 25, 33, 39, 40], psychological well-being and quality of life [37].

#### Self-esteem

**Fig. 8:**
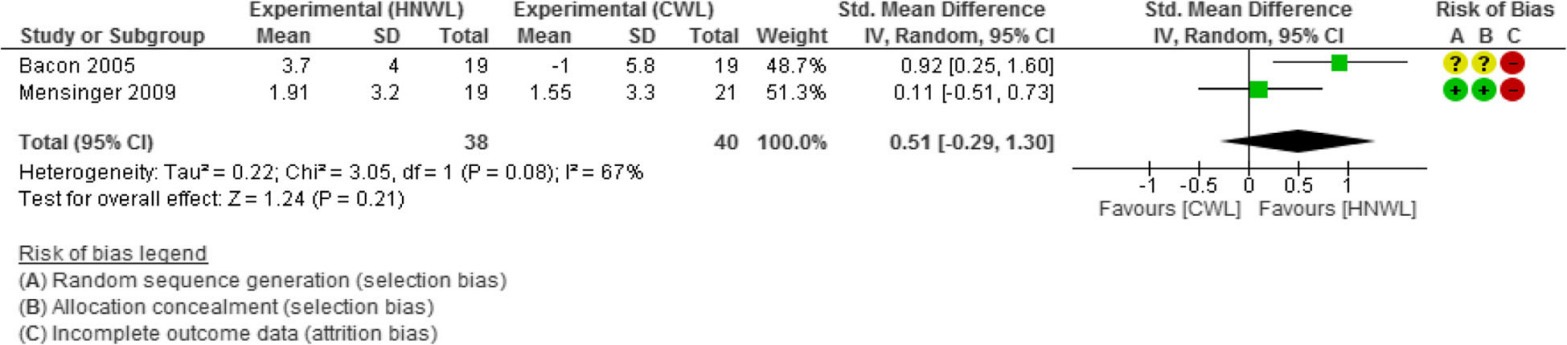
Meta-analysis of the results self-esteem at 53–104 weeks

This was measured using two scales across studies: the first was the state self-esteem scale (SSES), which gives a score between 54 and 83, with a high score indicating better self-esteem [39]; the second scale used was the Rosenberg self-esteem scale, where scores range between 10 and 40; with higher scores indicating higher self-esteem [23, 25]. The standardized mean difference in improvements in participants’ self-esteem was in favour of CWL programmes over HNWL programmes by 0.03 between weeks 8 and 19. It favoured the HNWL programmes by a standardized mean difference of 0.02 between weeks 20 and 39 and 0.17 at weeks 40 and 52. Low-moderate quality of evidence showed greater improvement in self-esteem on the HNWL programme during the longer follow-up period from weeks 53 to 104 (0.51 [− 0.29, 1.30] (Fig. 8)). This meta-analysis contained heterogeneity of 67% (*p* = 0.08) (Fig. 8), but this was considered moderate and below our a priori protocol threshold of 75% for random effects modelling.

#### Body image avoidance

This showed greater improvement on the HNWL programmes than CWL programmes at all stages of the treatment and the follow-up periods. The magnitude of the difference at weeks 8 to 19, showed a reduction in the HNWL programme by 3.7 points on the body-image avoidance questionnaire (BIAQ). This scale ranges from 1 to 74, and a greater score indicates a higher occurrence of body image avoidance. On average, overweight women score approximately 32 points. During the 20–39 week period, the HNWL programme showed a decrease by 4.8 points on the BIAQ. A 53–104 week follow-up the reduction in BIAQ favoured the HNWL programme by − 3.2 [− 8.34, 1.94] points, but this relied on data from just one study.

#### Depression

The Beck Depression Inventory (BDI) was used to measure depression; scores can range from 0 to 64 points. Greater scores reflect higher levels of depression on the BDI and a score of 12 is often used as an indication of clinical depression. Feelings of depression were reduced in both programmes over time, but there was less reduction in the HNWL programme, and therefore change in depression favoured the CWL programmes at all stages of the study by a greater reduction in score (mean difference − 0.83, 95% confidence interval [− 3.24, 1.57] at 8–19 weeks, − 0.26 [− 2.44 to 2.76] at 20–39 weeks, − 0.86 [− 3.47, 1.74] at 40–52 weeks and − 0.10 [− 5.19, 4.99] at 53–104 weeks), but this relied on data from just one study (see Additional file 2). Similar results were found when the enhanced programmes were removed from the analysis.

#### Binge eating

There was only one study containing data on binge eating from weeks 8 to 19 and weeks 40 to 52 and one study with results from weeks 53 to 104. These studies both reported changes in binge behaviour between weeks 20 and 39. At all stages, both groups showed a mean reduction in binge eating of between 5 and 14 on a scale with possible scores ranging between 0 and 65. A baseline value on the binge eating scale for both groups was 27, which is the threshold value indicative of bulimia nervosa. Participants on the CWL programmes showed a greater mean reduction than those on the HNWL programmes, but the difference between the groups was small (between 0 and 2).

#### Drive for thinness, bulimia and body dissatisfaction

These were measured using the Eating Disorder Inventory (EDI) [40] and EDI-2 [25, 39] scales and the body satisfaction scale [23].

#### Drive for thinness

The mean reduction at weeks 8 to 19 (− 1.10 [− 3.15 to 0.96]), weeks 20 to 39 (− 2.13 [− 3.93 to − 0.34]), weeks 42 to 52 (− 2.06 [− 4.02 to − 0.11]) (see Additional file 2) and, relying on data from just one study, weeks 53 to 104 (− 2.60 [− 5.11 to − 0.09]) were greater in the HNWL programme in comparison to the CWL programme. Removing the enhanced programme from the analysis showed a greater reduction in the HNWL programme at all stages in comparison to the CWL programmes.

#### Bulimia

The Eating Disorder Inventory (EDI) [40] and EDI-2 [25] scales were used to measure bulimia. The level of bulimia was reduced in both programmes, but the reduction was slightly greater in the CWL programme between weeks 8 to 19 (− 0.59 [− 1.73, 0.56]) (baseline values in both groups were 5) and at weeks 20 to 39 (− 0.04 [− 1.17, 1.09] (see Additional file 2). A reduction was reported across both programmes, but it was slightly greater in the HNWL programmes (− 0.07 [− 1.22 to 1.08] at weeks 40 to 52 and − 1.00 [− 2.95, 0.95] at weeks 53 to 104 and relied on one study’s data.

#### Body dissatisfaction

The baseline value in the studies that assessed body dissatisfaction using EDI was 22.2. One study assessed body satisfaction using BSS and baseline scores were 11. The standardised mean difference in change in body dissatisfaction favoured the HNWL programmes over the CWL programmes between weeks 8 and 19 (− 0.02 [− 0.33 to 0.29]), weeks 20 and 39 (− 0.22 [− 0.53 to 0.10]), weeks 40 and 52 (− 0.08 [− 0.42 to 0.26]), weeks 53 and104, (− 4.30 [− 8.32 to − 0.28]) but this was based on one study only. Sensitivity analysis removing enhanced programmes showed a similar pattern (available on request).

#### Hunger, disinhibition and restrained eating

These were measured using the three-factor eating questionnaire (TFEQ) [43]. This is a 51-item scale with each item scoring 0 or 1. Hunger has 14 items, disinhibition has 16 items and cognitive restraint has 21 items [23]. The minimum score for factors I-II-III is therefore 0-0-0 and the maximum possible score 21-16-14.

#### Hunger

Only two studies [23, 25] reported changes in hunger levels, showing a reduction in both programmes at all stages. The reduction was greater on the CWL programme at weeks 8 to 19 (− 0.56 [− 1.90 to 0.58]). On the HWNL programme, it was greater at weeks 20 to 39 (− 0.89 [− 2.23 to 0.45]) and weeks 40 to 52 (− 1.17 [− 2.48 to 0.13]) (see Additional file 2). Only one study [25] reported data from weeks 53 to 104 (− 1.20 [− 3.46 to 1.06]) which showed a greater reduction in hunger in the HNWL programme compared to CWL. The difference between the levels of reduction in hunger across the two programmes showed less than a 1.2 point variation on a scale of 0 to 14.

#### Disinhibition

Two studies reported disinhibition [23, 25], showing a reduction across both groups at all stages. The reduction was greater on the CWL programmes between weeks 8 to 19 by − 0.89 [− 2.16 to 0.37] and weeks 20 to 39 by − 0.20 [− 1.54 to 1.13]; it was greater on the HNWL programme from weeks 40 to 52 − 0.92 [− 2.28, 0.44] (available on request) and weeks 53 to 104 by − 2.30 [− 4.34 to − 0.26] but this relied on data from one study only [25]. The difference in disinhibition between the programmes therefore ranged between 0 and 2.3 on a scale of 0 to 16.

#### Restrained eating

There was a reduction in restrained eating behaviour in the HNWL programmes and an increase in the CWL programmes at all stages of the study. During this period, the reduction was of a magnitude between 5 and 12 on the restrained eating scale (0–21) and the difference between the programmes was between 3 and 9. Removing the enhanced study from the analysis showed that there was a greater reduction in the HNWL programme at all stages (available on request).

## Discussion

### Summary of main results

#### Primary outcomes

In the long-term follow-up period between weeks 53 and 104, changes to weight loss and total cholesterol-HDL ratio favoured the HNWL over the CWL programmes. The changes in blood pressure levels favoured the CWL programme. However, the differences between programmes for all of these primary outcomes were neither statistically significant nor of a mean magnitude which is of clinical consequence.

#### Secondary outcomes

Energy expenditure was slightly higher on the HNWL programme at all stages. Energy intake was reduced in both programmes with negligible differences between them. In the long-term, improvements to self-esteem and body image favoured the HNWL programme. The differences between programmes were minute. Feelings of depression and episodes of binge eating demonstrated a mean reduction in both programmes and favoured the CWL programme at all stages, but the differences were negligible. The HNWL programme showed greater reduction in disordered eating behaviours compared to the CWL programmes and in the drive for thinness and body dissatisfaction at all stages. The reduction in the bulimia subscale was greater in the HNWL programme in the longer term. In the long-term, the HNWL group showed a slightly greater reduction in the hunger factor and the disinhibition factor of the TFEQ. The HNWL group also showed a reduction at all stages in restrained eating behaviour.

### Quality and applicability of evidence

Many of the outcomes showed little real difference between the programme types, these were neither statistically significant nor clinically important. This may be because many of the components and behaviour change techniques are similar across the interventions. However, universally, CWL programmes promote weight loss through dietary restriction and weight monitoring while HNWL programmes do not; it may be this that accounts for the improvements in HNWL seen, particularly in restrained eating behaviour and body dissatisfaction, with a mean improvement in the longer term of 22% for restrained eating behaviour and 20% for body dissatisfaction. It is possible that the HNWL interventions were less true to their philosophy because of the research process. The HNWL philosophy, states that the participants should not focus on weight loss and record weight change. However, individuals are weighed at the beginning and end of the programme for gathering research data. This may have been a positive factor for achieving weight loss in the HNWL programmes in these studies, which might not have otherwise been found in a non-research setting.

There were no included studies that contained all outcomes of interest. Many of the studies had more women participants and seven of the eight studies were exclusively women; this limits the applicability of their results on a mixed population. Studies with a long follow-up period of more than 2 years were not available. RCTs showed that the HNWL programme was more successful than CWL in the long-term but as this was up to 2 years of follow-up more studies that specifically compare the two programmes over a longer period of time are needed before results can be certain.

The risk of bias in some of the included trials was high, with six of the eight studies not identifying how random allocation sequences and concealment were administered. Furthermore, many studies showed a high risk of attrition bias. Considering only those studies with a lower risk of bias and removing those studies with enhanced elements reduced the sample size of studies and in some cases the results from a single study were relied on. However, these sensitivity and sub-analyses made little difference to the direction of the findings and the interpretation of them.

### Strengths and limitations with respect to other studies

There is no existing meta-analysis of RCTs conducted in this area, of the two systematic reviews published to date, one combined results from a variety of study designs [28] and the other included a variety of control groups [29].

Schaefer’s [28] study examined the effects of intuitive eating. It included 24 articles on 20 different studies and nine were randomised controlled trials. Only two of these studies were included in our research [25, 40] because the others did not use CWL as a control group. The studies showed improvements in eating habits, lifestyle and body image and improved psychological health. Several improvements were sustained throughout the follow-up periods for as long as 2 years. One prospective cohort study [44] followed participants for 3 years and found that participants maintained increased physical activity and self-esteem and a decrease in restrained eating. In one RCT [45], 14 participants maintained intuitive eating habits after 10 years. However, the lack of a conventional weight loss control group in these studies limits the confidence we can put in the results as a potential alternative to conventional care.

Similarly, Clifford et al.’s [29] systematic review included studies which were both quasi-experimental and randomised designs where the control was not conventional weight loss. Therefore only four of the studies included in the Clifford review [23, 25, 36, 40] matched our strict inclusion criteria of precise definitions for conventional weight loss controls and HNWL interventions (which did not have weight loss targets). Our broader search terms and more recent search of the databases led to inclusion of additional trials, providing us with more up to date and comprehensive results.

Clifford et al. showed statistically significant improvements in disordered eating behaviour, depression and self-esteem. They found no significant weight gain or raised blood pressure, blood glucose or cholesterol caused by these interventions. In two of the studies, biochemical measures were improved. However, because of the broad inclusion criteria, these results could not be pooled in a meta-analysis and the effects at different time-points are unclear.

We found that biochemical and weight parameters were better with CWL programmes in the short term, but in the long term there were no significant differences, so only in the long term were our results consistent with Clifford et al. Clifford et al. found significant improvements in depression, we only found long-term significant improvements, beyond that of conventional programmes, in restrained eating behaviour and body satisfaction; these effects were small and relied on data from a single study. We recommend large non-inferiority trials are needed between HNWL and CWL programmes to confirm these conclusions.

## Conclusions

The effects of HNWL programmes compared to CWL programmes show no long term, significant differences in blood lipids, hypertension and weight loss. This is consistent with previous systematic reviews. However, losses to follow up in some studies were high and the estimates resulting from meta-analyses were often imprecise. HNWL programmes were slightly better at long-term improvement in disordered eating behaviour and body satisfaction, but results were often drawn from a single study. Therefore large, long-term, high-quality randomised controlled trials are now needed to confirm our findings before firm clinical recommendations can be made regarding the comparative non-inferiority or superiority of these programmes.

## Additional files

Additional file 1: Search strategies. (DOCX 31 kb)

Additional file 2: Additional Meta-analysis. (DOCX 975 kb)

## Data Availability

All data generated or analysed during this study are included in this published article and its supplementary information files.

## Abbreviations

BMI: Body mass index
CI: Confidence interval
CWL: Conventional weight loss
HAES: Health at every size
HNWL: Health, not weight loss, focused
IE: Intuitive eating
MD: Mean difference
RCT: Randomised control trials
RR: Relative risk
