# Supplementary material for: Health, not weight loss, focused programmes versus conventional weight loss programmes for cardiovascular risk factors: a systematic review and meta-analysis
Source: Syst Rev. 2019 Aug 10;8:200. doi: 10.1186/s13643-019-1083-8 (PMC6689181; doi:10.1186/s13643-019-1083-8)
Supplement: Supplementary file 1 — Search strategies. (DOCX 31 kb) [file 13643_2019_1083_MOESM1_ESM.docx]

**Additional File 1: Search Strategies**

**Cochrane Central Register of Controlled Trials Searched between 1960 and 3 March 2019**

1. "health at every size"
2. HAES
3. (weight adj4 (manag* or accept* or centred*or centered*))
4. (health* adj6 (size or weigh*))
5. "Non diet*"
6. "non-diet*"
7. "nondiet*"
8. ((Wholistic or holistic) adj4 weight)
9. ((Intuitiv* or attentiv* or mindful*) adj4 eat*)
10. #1 OR #2 OR #3 OR #4 OR #5 OR #6 OR #7 OR #8 OR #9
11. Hypertension
12. hypertensi*
13. blood pressure*
14. Blood Pressure
15. Cholesterol
16. cholesterol*
17. Cholesterol, HDL
18. Cholesterol, LDL
19. Triglycerides
20. triglyceride*
21. triacylglycerol*
22. lipoprotein*
23. bmi
24. overweight
25. body mass index
26. Abdominal Fat
27. Overweight
28. obes*
29. Obesity
30. (weight adj2 (gain* or chang* or los* or maint*))
31. (body mass adj (index or indexes or indices))
32. abdominal fat
33. quetelet*
34. ((reduc* or increas* or decreas* or los* or gain*) adj2 weight)
35. Body fat
36. Fat mass
37. Waist
38. Diet*
39. Intake
40. Consumption
41. food
42. beverages
43. drinking behavior
44. (physical adj activit*)
45. exercise
46. (physical adj activit*)
47. Affective symptoms
48. depression
49. stress
50. psychological
51. well-being
52. Binge*
53. Three-factor
54. Disinhibition
55. Hunger
56. Restraint
57. Psychological
58. stress
59. (Body adj (image or satisfaction or dissatisfaction))
60. #11 OR #12 OR #13 OR #14 OR #15 OR #16 OR #17 OR #18 OR #19 OR #20 OR #21 OR #22 OR #23 OR #24 OR #25 OR #26 OR #27 OR #28 OR #29 OR #30 OR #31 OR #32 OR #33 OR #34 OR #35 OR #36 OR #37 OR #38 OR #39 OR #40 OR #41 OR #42 OR #43 OR #44 OR #45 OR #46 OR #47 OR #48 OR #49 OR #50 OR #51 OR #52 OR #53 OR #54 OR #55 OR #56 OR #57 OR #58 OR #59
61. (randomized controlled trial):PT
62. (controlled clinical trial):PT
63. randomized:AB
64. placebo:AB
65. drug therapy
66. randomly:AB
67. trial:AB
68. groups:AB
69. #61 OR #62 OR #63 OR #64 OR #65 OR #66 OR #67 OR #68
70. animals
71. humans
72. #70 NOT #71
73. #69 NOT #72
74. #10 AND #60
75. #73 AND #74

**CINAHL 1981-5 April 2019**

**1981 to Aug 2015 (OVID)**

| 1. TX "health at every size" |
| --- |
| 1. TX HAES |
| 1. TX weight pre4 manag* |
| 1. TX weight pre4 accept* |
| 1. TX weight pre4 centred* |
| 1. TX weight pre4 centered* |
| 1. TX health* pre6 size |
| 1. TX health* pre6 weigh* |
| 1. TX “Non diet*” |
| 1. TX “non-diet*” |
| 1. TX Wholistic pre4 weight |
| 1. TX holistic pre4 weight |
| 1. TX Intuitiv* pre4 eat* |
| 1. TX attentiv* pre4 eat* 2. TX mindful* pre4 eat* 3. S1 OR S2 OR S3 OR S4 OR S5 OR S6 OR S7 OR S8 OR S9 OR S10 OR S11 OR S12 OR S13 OR S14 OR S15 4. SU Hypertension/ |
| 1. TX hypertensi* |
| 1. TX blood pressure |
| 1. SU Blood Pressure/ |
| 1. SU Cholesterol/ |
| 1. TX cholesterol* |
| 1. SU Cholesterol, HDL/ |
| 1. SU Cholesterol, LDL/ |
| 1. SU Triglycerides/ |
| 1. TX triglyceride* |
| 1. TX triacylglycerol* |
| 1. TX lipoprotein* |
| 1. TX bmi |
| 1. TX overweight |
| 1. SU body mass index/ |
| 1. SU Abdominal Fat/ |
| 1. SU Overweight/ |
| 1. TX obes* |
| 1. SU Obesity/ |
| 1. TX weight pre2 gain* |
| 1. TX weight pre2 chang* |
| 1. TX weight pre2 los* |
| 1. TX weight pre2 maint* |
| 1. TX body mass pre index |
| 1. TX body mass pre indexes |
| 1. TX body mass pre indices |
| 1. TX abdominal fat |
| 1. TX quetelet* index |
| 1. TX reduc* pre2 weight |
| 1. TX increas* pre2 weight |
| 1. TX decreas* pre2 weight |
| 1. TX los* pre2 weight |
| 1. TX gain* pre2 weight |
| 1. TX Body fat |
| 1. TX Fat mass |
| 1. TX Waist |
| 1. TX Diet* |
| 1. TX Intake |
| 1. TX Consumption |
| 1. SU food/ |
| 1. SU beverages/ |
| 1. SU drinking behavior/ |
| 1. TX physical pre activit* |
| 1. SU exercise/ |
| 1. SU Affective symptoms/ |
| 1. SU depression/ |
| 1. SU stress, psychological/ |
| 1. TX well-being |
| 1. TX Binge* |
| 1. TX Three-factor |
| 1. TX Disinhibition |
| 1. TX Hunger |
| 1. TX Restraint |
| 1. TX Body pre image |
| 1. TX Body pre satisfaction |
| 1. TX Body pre dissatisfaction |
| 1. S17 OR S18 OR S19 OR S20 OR S21 OR S22 OR S23 OR S24 OR S25 OR S26 OR S27 OR S28 OR S29 OR S30 OR S31 OR S32 OR S33 OR S34 OR S35 OR S36 OR S37 OR S38 OR S39 OR S40 OR S41 OR S42 OR S43 OR S44 OR S45 OR S46 OR S47 OR S48 OR S49 OR S50 OR S51 OR S52 OR S53 OR S54 OR S55 OR S56 OR S57 OR S58 OR S59 OR S60 OR S61 OR S62 OR S63 OR S64 OR S65 OR S66 OR S67 OR S68 OR S69 OR S70 OR S71 OR S72 |
| 1. PT randomized controlled trial |
| 1. PT controlled clinical trial |
| 1. AB randomized |
| 1. AB placebo |
| 1. MJ drug therapy |
| 1. AB randomly |
| 1. AB trial |
| 1. AB groups |
| 1. S74 OR S75 OR S76 OR S77 OR S78 OR S79 OR S80 OR S81 |
| 1. SU animals/ NOT SU humans |
| 1. S82 not S83 |
| 1. S16 and s73 |
| 1. S84 AND S85   **Aug 2015-5 April 2019 (Ebscohost)** |

1. TX "health at every size"

2. TX HAES

3. TX weight manag*

4. TX weight accept*

5. TX weight centred*

6. TX weight centered*

7. TX health at (every or any) size

8. TX “Non diet*”

9. TX “non-diet*”

10. TX Intuitiv* eat* or eat* Intuitiv*

11. TX attentiv* eat* or eat* attentiv*

12. TX mindful* eat* or eat* indful*

13. SU Hypertension/

14. TX hypertensi*

15. TX blood pressure

16. SU Blood Pressure/

17. SU Cholesterol/

18. TX cholesterol*

19. SU Cholesterol, HDL/

20. SU Cholesterol, LDL/

21. SU Triglycerides/

22. TX triglyceride*

23. TX triacylglycerol*

24. TX lipoprotein*

25. TX bmi

26. TX overweight

27. SU body mass index/

28. SU Abdominal Fat/

29. TX obes*

30. SU Obesity/

31. TX weight gain*

32. TX weight Chang*

33. TX weight los*

34. TX weight maint*

35. TX body mass pre index

36. TX body mass indexes

37. TX body mass indices

38. TX abdominal fat

39. TX quetelet* index

40. TX reduc* weight

41. TX increas* weight

42. TX decreas* weight

43. TX los* weight

44. TX gain* weight

45. TX Body fat

46. TX Fat mass

47. TX Waist

48. TX Diet*

49. TX Intake

50. TX Consumption

51. SU food/

52. SU beverages/

53. SU drinking behavior/

54. TX physical pre activit*

55. SU exercise/

56. SU Affective symptoms/

57. SU depression/

58. SU stress, psychological/

59. TX well-being

60. TX Binge*

61. TX Three-factor

62. TX Disinhibition

63. TX Hunger

64. TX Restraint

65. TX Body image

66. TX Body satisfaction

67. TX Body dissatisfaction

68. SU animals/ NOT SU humans

69. S1 OR S2 OR S3 OR S4 OR S5 OR S6 OR S7 OR S8 OR S9 OR S10 OR S11 OR S12

70. S13 OR S14 OR S15 OR S16 OR S17 OR S18 OR S19 OR S20 OR S21 OR S22 OR S23 OR S24 OR S25 OR S26 OR S27 OR S28 OR S29 OR S30 OR S31 OR S32 OR S33 OR S34 OR S35 OR S36 OR S37 OR S38 OR S39 OR S40 OR S41 OR S42 OR S43 OR S44 OR S45 OR S46 OR S47 OR S48 OR S49 OR S50 OR S51 OR S52 OR S53 OR S54 OR S55 OR S56 OR S57 OR S58 OR S59 OR S60 OR S61 OR S62 OR S63 OR S64 OR S65 OR S66 OR S67

71. S69 AND S70

72. s71 NOT s68

73. AB random*

74. AB weight

75. S74 AND S75

76. S73 AND S76

**MEDLINE: 1946-5 April 2019**

**1946 to 3 August 2015 (OVID)**

1. "health at every size".tw.

2. HAES.tw.

3. (weight adj4 (manag* or accept* or centred*or centered*)).tw.

4. (health* adj6 (size or weigh*)).tw.

5. "Non diet*".tw.

6. "non-diet*".tw.

7. "nondiet*".tw.

8. ((Wholistic or holistic) adj4 weight).tw.

9. ((Intuitiv* or attentiv* or mindful*) adj4 eat*).tw.

10. or/1-9

11. exp Hypertension/

12. hypertensi*.tw.

13. blood pressure.tw.

14. Blood Pressure/

15. Cholesterol/

16. cholesterol*.tw.

17. Cholesterol, HDL/

18. Cholesterol, LDL/

19. Triglycerides/

20. triglyceride*.tw.

21. triacylglycerol*.tw.

22. lipoprotein*.tw.

23. bmi.tw.

24. overweight.tw.

25. body mass index/

26. exp Abdominal Fat/

27. exp Overweight/

28. obes*.tw.

29. exp Obesity/

30. (weight adj2 (gain* or chang* or los* or maint*)).tw.

31. (body mass adj (index or indexes or indices)).tw.

32. abdominal fat.tw.

33. quetelet* index.tw.

34. ((reduc* or increas* or decreas* or los* or gain*) adj2 weight).tw.

35. Body fat.tw.

36. Fat mass.tw.

37. Waist.tw.

38. Diet*.tw.

39. Intake.tw.

40. Consumption.tw.

41. exp food/ and beverages/

42. exp drinking behavior/

43. (physical adj activit*).tw.

44. exp exercise/

45. exp Affective symptoms/

46. exp depression/

47. exp stress, psychological/

48. well-being.tw.

49. Binge*.tw.

50. Three-factor.tw.

51. Disinhibition.tw.

52. Hunger.tw.

53. Restraint.tw.

54. (Psychological adj $stress).tw.

55. (Body adj (image or satisfaction or dissatisfaction)).tw.

56. or/11-55

57. randomized controlled trial.pt.

58. controlled clinical trial.pt.

59. randomized.ab.

60. placebo.ab.

61. drug therapy.fs.

62. randomly.ab.

63. trial.ab.

64. groups.ab.

65. 57 or 58 or 59 or 60 or 61 or 62 or 63 or 64

66. exp animals/ not humans.sh.

67. 65 not 66

68. 10 and 56

69. 67 and 68

70. limit 69 to yr="1970 -Current"

**3 August 2015-5 April 2019 (EBSCOhost)**

1. TX "health at every size"
2. TX HAES
3. TX (weight (manag* or accept* or centred*or centered*))
4. TX (health at (any or every) size
5. TX "Non diet*"
6. TX "non-diet*"
7. TX "nondiet*"
8. TX ((Intuitiv* or attentiv* or mindful*) eat*)
9. exp Hypertension/
10. TX hypertensi*
11. TX blood pressure
12. TX blood pressure
13. blood pressure
14. cholesterol
15. TX cholesterol*
16. Cholesterol, HDL/
17. Cholesterol, LDL/
18. triglycerides
19. TX triglyceride*
20. TX triacylglycerol*
21. TX lipoprotein*
22. TX bmi
23. TX overweight
24. body mass index
25. exp Abdominal Fat/
26. exp Overweight/
27. TX obes*
28. exp Obesity/
29. TX (weight (gain* or chang* or los* or maint*))
30. TX (body mass adj (index or indexes or indices))
31. TX abdominal fat
32. TX quetelet* index
33. TX ((reduc* or increas* or decreas* or los* or gain*) weight)
34. TX Body fat
35. TX Fat mass
36. TX Waist
37. TX Diet*
38. TX Intake
39. TX Consumption
40. exp food/ and beverages/
41. exp drinking behavior/
42. TX (physical adj activit*)
43. exp exercise/
44. exp Affective symptoms/
45. exp depression/
46. exp stress, psychological/
47. TX well-being
48. TX Binge*
49. TX Three-factor
50. TX Disinhibition
51. TX Hunger
52. TX Restraint
53. TX (Psychological adj $stress)
54. TX (Body adj (image or satisfaction or dissatisfaction))
55. S1 OR S2 OR S3 OR S4 OR S5 OR S6 OR S7 OR S8
56. S9 OR S10 OR S11 OR S12 OR S13 OR S14 OR S15 OR S16 OR S17 OR S18 OR S19 OR S20 OR S21 OR S22 OR S23 OR S24 OR S25 OR S26 OR S27 OR S28 OR S29 OR S30 OR S31 OR S32 OR S33 OR S34 OR S35 OR S36 OR S37 OR S38 OR S39 OR S40 OR S41 OR S42 OR S43 OR S44 OR S45 OR S46 OR S47 OR S48 OR S49 OR S50 OR S51 OR S52 OR S53 OR S54
57. S55 AND S56
58. **Limiters** - Date of Publication: 20150801-20190331; English Language; Human; Publication Type: Clinical Trial, Clinical Trial, Phase I, Clinical Trial, Phase II, Clinical Trial, Phase III, Clinical Trial, Phase IV, Controlled Clinical Trial, Randomized Controlled Trial
59. S57 AND S58
60. AB weight
61. AB random*
62. S59 AND S60 AND S61

**PsychINFO**

**Ovid: 1967 to Aug 2015**

1. "health at every size".tw.

2. HAES.tw.

3. (weight adj4 (manag* or accept* or centred*or centered*)).tw.

4. (health* adj6 (size or weigh*)).tw.

5. "Non diet*".tw.

6. "non-diet*".tw.

7. "nondiet*".tw.

8. ((Wholistic or holistic) adj4 weight).tw.

9. ((Intuitiv* or attentiv* or mindful*) adj4 eat*).tw.

10. or/1-9

11. exp Hypertension/

12. hypertensi*.tw.

13. blood pressure.tw.

14. Blood Pressure/

15. Cholesterol/

16. cholesterol*.tw.

17. exp Lipoproteins/

18. Triglycerides.mp.

19. triacylglycerol*.tw.

20. lipoprotein*.tw.

21. bmi.tw.

22. overweight.tw.

23. body mass index/

24. exp body Fat/

25. exp Overweight/

26. obes*.tw.

27. exp Obesity/

28. (weight adj2 (gain* or chang* or los* or maint*)).tw.

29. (body mass adj (index or indexes or indices)).tw.

30. abdominal fat.tw.

31. quetelet* index.tw.

32. ((reduc* or increas* or decreas* or los* or gain*) adj2 weight).tw.

33. Body fat.tw.

34. Fat mass.tw.

35. Waist.tw.

36. Diet*.tw.

37. Intake.tw.

38. Consumption.tw.

39. exp food/

40. exp food intake/

41. exp alcoholic beverages/

42. exp "Beverages (Nonalcoholic)"/

43. exp drinking behavior/

44. (physical adj activit*).tw.

45. exp exercise/

46. exp Affective Psychosis/

47. exp depression/

48. exp Psychological Stress/

49. well-being.tw.

50. Binge*.tw.

51. Three-factor.tw.

52. Disinhibition.tw.

53. Hunger.tw.

54. Restraint.tw.

55. (Psychological adj $stress).tw.

56. (Body adj (image or satisfaction or dissatisfaction)).tw.

57. or/11-56

58. exp clinical trial/

59. randomized.ab.

60. placebo.ab.

61. *drug therapy/ or exp clinical trials/

62. randomly.ab.

63. trial.ab.

64. groups.ab.

65. 58 or 59 or 60 or 61 or 62 or 63 or 64

66. exp animals/ not humans.sh.

67. 65 not 66

68. 10 and 57

69. 67 and 68

70. limit 69 to yr="1970 -Current"

**Aug 2015-5 April 2019 (EBSCOhost)**

1. TX "health at every size"

2. TX HAES

3. TX (weight (manag* or accept* or centred*or centered*))

4. TX (health* (size or weigh*))

5. TX "Non diet*"

6. TX "non-diet*"

7. T TX ((Wholistic or holistic) weight) X "nondiet*"

8. TX ((Intuitiv* or attentiv* or mindful*) eat*)

9. S1 OR S2 OR S3 OR S4 OR S5 OR S6 OR S7 OR S8 OR S9

10. DE Hypertension

11. blood pressure

12. cholesterol

13. TX cholesterol*

14. DE Lipoproteins

15. Triglycerides

16. TX triacylglycerol*

17. TX lipoprotein*

18. TX bmi

19. TX overweight

20. TX body mass index/

21. body mass index

22. DE body Fat

23. DE Overweight

24. TX obes*

25. DE Obesity

26. TX (weight (gain* or chang* or los* or maint*))

27. TX (body mass (index or indexes or indices))

28. TX abdominal fat

29. TX quetelet* index

30. TX ((reduc* or increas* or decreas* or los* or gain*) weight)

31. TX Body fat

32. TX Fat mass

33. TX Waist

34. TX Diet*

35. TX Intake

36. TX Consumption

37. DE food

38. DE food intake

39. DE alcoholic beverages

40. DE "Beverages (Nonalcoholic)"

41. ED drinking behavior

42. TX (physical activit*)

43. DE exercise

44. DE Affective Psychosis

45. DE depression

46. DE Psychological Stress

47. TX well-being

48. TX Binge*

49. TX Three-factor

50. TX Disinhibition

51. TX Hunger

52. TX Restraint

53. TX (Psychological stress)

54. TX (Body (image or satisfaction or dissatisfaction))

55. S11 OR S12 OR S13 OR S14 OR S15 OR S16 OR S17 OR S18 OR S19 OR S20 OR S21 OR S22 OR S23 OR S24 OR S25 OR S26 OR S27 OR S28 OR S29 OR S30 OR S31 OR S32 OR S33 OR S34 OR S35 OR S36 OR S37 OR S38 OR S39 OR S40 OR S41 OR S42 OR S43 OR S44 OR S45 OR S46 OR S47 OR S48 OR S49 OR S50 OR S51 OR S52 OR S53 OR S54 OR S55

56. S10 AND S56

57. Limiters - English; Population Group: Human; Methodology: CLINICAL TRIAL

58. Limiters - Published Date: 20150801-20190331

59. S58 AND S59

60. S57 AND S60

EMBASE

1974 to Aug 2015 (OVID)

1. "health at every size".tw.

2. HAES.tw.

3. (weight adj4 (manag* or accept* or centred*or centered*)).tw.

4. (health* adj6 (size or weigh*)).tw.

5. "Non diet*".tw.

6. "non-diet*".tw.

7. "nondiet*".tw.

8. ((Wholistic or holistic) adj4 weight).tw.

9. ((Intuitiv* or attentiv* or mindful*) adj4 eat*).tw.

10. or/1-9

11. exp Hypertension/

12. hypertensi*.tw.

13. blood pressure.tw.

14. Blood Pressure/

15. Cholesterol/

16. cholesterol*.tw.

17. Cholesterol, HDL/

18. Cholesterol, LDL/

19. Triglycerides/

20. triglyceride*.tw.

21. triacylglycerol*.tw.

22. lipoprotein*.tw.

23. bmi.tw.

24. overweight.tw.

25. body mass index/

26. exp Abdominal Fat/

27. exp Overweight/

28. obes*.tw.

29. exp Obesity/

30. (weight adj2 (gain* or chang* or los* or maint*)).tw.

31. (body mass adj (index or indexes or indices)).tw.

32. abdominal fat.tw.

33. quetelet* index.tw.

34. ((reduc* or increas* or decreas* or los* or gain*) adj2 weight).tw.

35. Body fat.tw.

36. Fat mass.tw.

37. Waist.tw.

38. Diet*.tw.

39. Intake.tw.

40. Consumption.tw.

41. exp food/ and beverages/

42. exp drinking behavior/

43. (physical adj activit*).tw.

44. exp exercise/

45. exp Affective symptoms/

46. exp depression/

47. exp stress, psychological/

48. well-being.tw.

49. Binge*.tw.

50. Three-factor.tw.

51. Disinhibition.tw.

52. Hunger.tw.

53. Restraint.tw.

54. (Psychological adj $stress).tw.

55. (Body adj (image or satisfaction or dissatisfaction)).tw.

56. or/11-55

57. clinical trial/

58. controlled study/

59. randomized.ab.

60. placebo.ab.

61. drug therapy/

62. randomly.ab.

63. trial.ab.

64. groups.ab.

65. 57 or 58 or 59 or 60 or 61 or 62 or 63 or 64

66. exp animals/ not humans.sh.

67. 65 not 66

68. 10 and 56

69. 67 and 68

70. limit 69 to yr="1970 -Current"

**ASSIA (Proquest)**

**1987 to 5 April 2019**

| 1. (health at every size) OR (health at any size) |
| --- |
| 1. HAES |
| 1. (weight management) OR (adult weight management) OR (managing weight) OR (healthy weight management) |
| 1. Weight centred |
| 1. Weight manag* |
| 1. weight accept* |
| 1. Non diet* |
| 1. Non diet* |
| 1. holistic weight |
| 1. wholistic weight |
| 1. (mindful eat*) OR (eat* mindful*) |
| 1. (intuitive eat*) OR (eat* intuitiv*) |
| 1. intuitive eating research |
| 1. health* size |
| 1. health* weight |
| 1. S1 or S2 or S3 or S4 or S5 or S6 or S7 or S8 or S9 or S10 or S11 or S12 or S13 or S14 or S15 |
| 1. hypertensi* |
| 1. blood pressure |
| 1. cholesterol* |
| 1. (high density) OR (high density lipids) |
| 1. triglycerid* |
| 1. triacylglycerol* |
| 1. *triglyceride |
| 1. *triacylglycerol |
| 1. lipoprotein* |
| 1. bmi |
| 1. (body mass index) OR (lean body mass) OR (body mass) OR (body mass index AND body image) OR (body mass body image dieting) OR (beyond body mass index) |
| 1. overweight |
| 1. abdominal fat |
| 1. obes* |
| 1. weight gain* |
| 1. gain weight |
| 1. weight chang* |
| 1. weight los* |
| 1. (weight maint*) OR (maint* weight) |
| 1. lose weigh |
| 1. quetelet* index |
| 1. reduc* weight |
| 1. (reduce weight) OR (reduce fat) |
| 1. increas* weight |
| 1. decreas* weight |
| 1. los* weight |
| 1. gain* weight |
| 1. Body fat |
| 1. Fat mass |
| 1. waist |
| 1. Diet* |
| 1. intake |
| 1. consumption |
| 1. food* |
| 1. beverages OR (a beverage) OR (diet beverage) |
| 1. drinking behavior* |
| 1. physical PRE/4 activit* |
| 1. exercise |
| 1. Affective symptoms |
| 1. depression |
| 1. abdominal |
| 1. quetelet |
| 1. weight gain |
| 1. psychological stress |
| 1. (well being) OR (well AND being) OR (well- being) |
| 1. Binge* |
| 1. Three-factor |
| 1. Disinhibition |
| 1. Hunger |
| 1. Restraint |
| 1. Psychological PRE/4 *stress |
| 1. Body PRE/4 image |
| 1. Body PRE/4 satisfaction |
| 1. Body PRE/4 dissatisfaction |
| 1. S17 or S18 or S19 or S20 or S21 or S22 or S23 or S24 or S25 or S26 or S27 or S28 or S29 or S30 or S31 or S32 or S33 or S34 or S35 or S36 or S37 or S38 or S39 or S40 or S41 or S42 or S43 or S44 or S45 or S46 or S47 or S48 or S49 or S50 or S51 or S52 or S53 or S54 or S55 or S56 or S57 or S58 or S59 or S60 or S61 or S62 or S63 or S64 or S65 or S66 or S67 or S68 or S69 or S70 |
| 1. (a randomized controlled) OR (randomized control study) OR (randomized control trial) OR (randomized controlled) OR (a randomized controlled trial) OR (randomized controlled trial) |
| 1. controlled clinical trial |
| 1. randomized |
| 1. placebo |
| 1. drug therapy |
| 1. S72 or S73 or S74 or S75 or S76 |
| 1. animals |
| 1. humans |
| 1. S78 NOT S79 |
| 1. S77 NOT S80 |
| 1. S16 AND S71 |
| 1. S81 AND S82 |
