# Supplementary material for: Health, not weight loss, focused programmes versus conventional weight loss programmes for cardiovascular risk factors: a systematic review and meta-analysis
Source: Syst Rev. 2019 Aug 10;8:200. doi: 10.1186/s13643-019-1083-8 (PMC6689181; doi:10.1186/s13643-019-1083-8)
Supplement: Supplementary file 2 — Additional Meta-analysis. (DOCX 975 kb) [file 13643_2019_1083_MOESM2_ESM.docx]

**Additional File 2: Additional Meta-analysis**

**Results for total cholesterol**

The mean difference [95% confidence interval] in effect on cholesterol levels was in favour of the CWL (-0.32mmol/L [-0.01 to - 0.64]) compared to the HNWL programmes at the end of treatment (Figure 1) and (0.06 mmoI/L [-0.37 to 0.25] weeks 40-52 ([Figure](file:///C:\Users\ab5042\AppData\Local\Microsoft\Windows\Temporary%20Internet%20Files\Content.Outlook\L63T0RLL\001.03) 3). The reduction in total cholesterol was slightly greater in the HNWL programmes (0.05 mmoI/L [-0.31 to 0.21]), compared to the CWL programmes at weeks 20 to 39 (Figure 2). During the longer follow-up period between weeks 53 to 104 showed that improvements in total cholesterol were slightly greater in the HNWL programmes (- 0.17mmol/L [-0.51 to 0.18]) ([Figure](file:///C:\Users\ab5042\AppData\Local\Microsoft\Windows\Temporary%20Internet%20Files\Content.Outlook\L63T0RLL\001.04) 4) compared to CWL programmes.


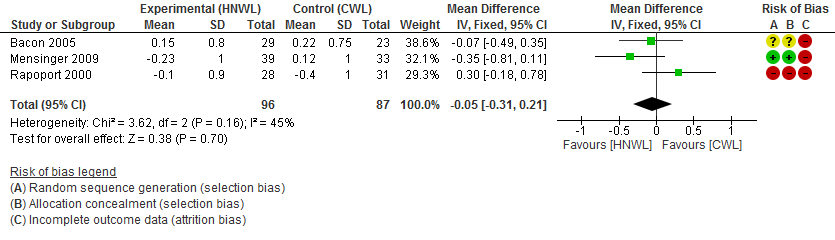
Figure 1. Meta-analysis for Cholesterol at 8–19 weeks. Figure 2. Meta-analysis for Cholesterol at 20–39 weeks.
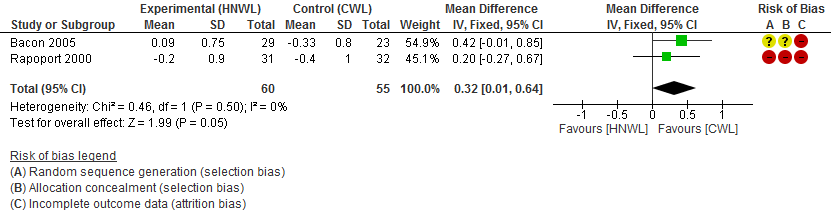


Figure 3. Meta-analysis for Cholesterol at 40–52 weeks Figure 4. Meta-analysis for Cholesterol at 53–104 weeks


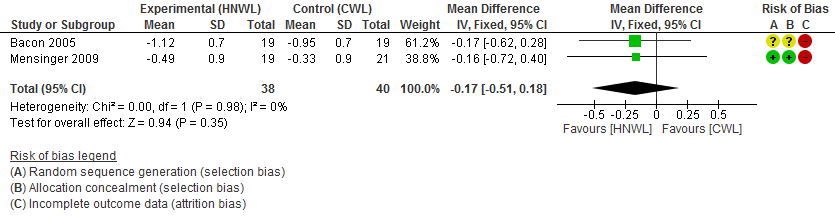

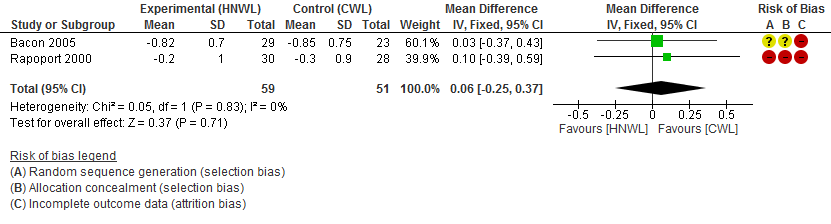


**Results for high-density lipoprotein (HDL)**

HDL reduced in both programmes, but was more favoured in the CWL programmes compared to the HNWL programmes at weeks 20 and 39, (<0.01mmoI/L[-0.07 to 0.08]) ([Figure](file:///C:\Users\ab5042\AppData\Local\Microsoft\Windows\Temporary%20Internet%20Files\Content.Outlook\L63T0RLL\002.02) 6) and between weeks 40 to 52 (0.02 mmoI/L [-009 to 0.13]) ([Figure](file:///C:\Users\ab5042\AppData\Local\Microsoft\Windows\Temporary%20Internet%20Files\Content.Outlook\L63T0RLL\002.03) 7). Between weeks 8-19 (0.04 mmoI/L [-0.13 to 0.06]) ([Figure](file:///C:\Users\user\Downloads\002.01) 5), and weeks 53 to 104 (0.02 mmoI/L [-0.10 to 0.05]) ([Figure](file:///C:\Users\user\Downloads\002.04) 8) the change in HDL levels was slighter more favourable in the HNWL programmes than the CWL programmes.

Figure 5. Meta-analysis for HDL at 8–19 weeks Figure 6. Meta-analysis for HDL at 20–39 weeks


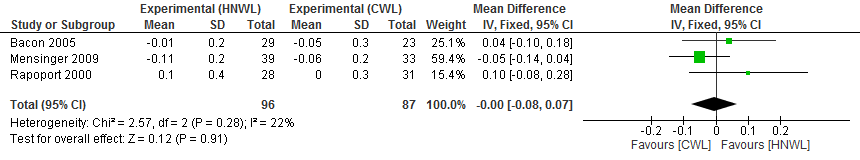


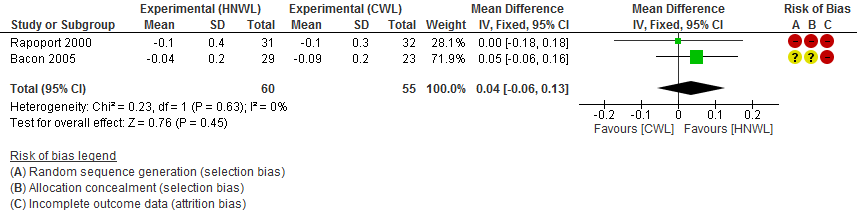


Figure 7. Meta-analysis for HDL at 40–52 weeks Figure 8. Meta-analysis for HDL at 53–104 weeks


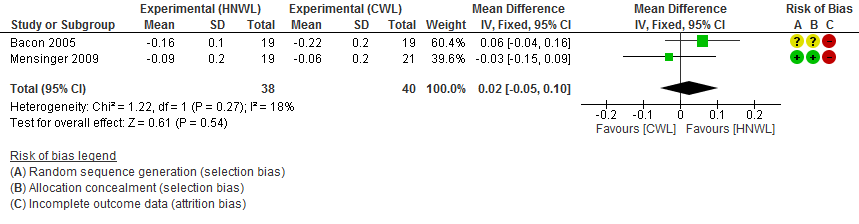

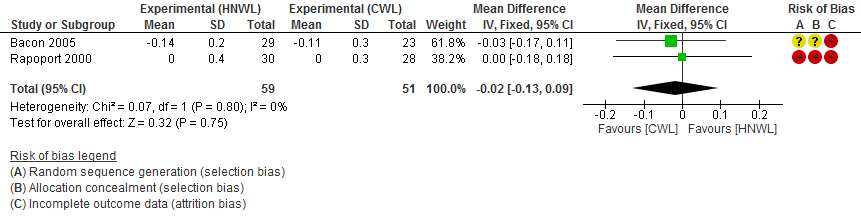


**Results for low-density lipoprotein (LDL)**

The mean differences in change in LDL were in favour of the CWL programmes compared to the HNWL programmes post treatment (- 0.22 mmoI/L [-0.48 to 0.04]) ([Figure](file:///C:\Users\ab5042\AppData\Local\Microsoft\Windows\Temporary%20Internet%20Files\Content.Outlook\L63T0RLL\003.01) 9), at weeks 20-39, (-0.08 mmoI/L[-0.30 to 0.13]) ([Figure](file:///C:\Users\ab5042\AppData\Local\Microsoft\Windows\Temporary%20Internet%20Files\Content.Outlook\L63T0RLL\003.02) 10) and weeks 40 to 52 (-0.09 mmoI/L[-0.35 to 0.18 ]) ([Figure](file:///C:\Users\ab5042\AppData\Local\Microsoft\Windows\Temporary%20Internet%20Files\Content.Outlook\L63T0RLL\003.03) 11) Whereas the change in LDL was in favour of the HNWL programmes than the CWL participants during the longer follow-up period (-0.20 mmoI/L[-0.44 to 0.04]) (weeks 53 to 104) ([Figure](file:///C:\Users\ab5042\AppData\Local\Microsoft\Windows\Temporary%20Internet%20Files\Content.Outlook\L63T0RLL\003.04) 12).

Figure 9. Meta-analysis for LDL at 8–19 weeks Figure 10. Meta-analysis for LDL at 20–39 weeks


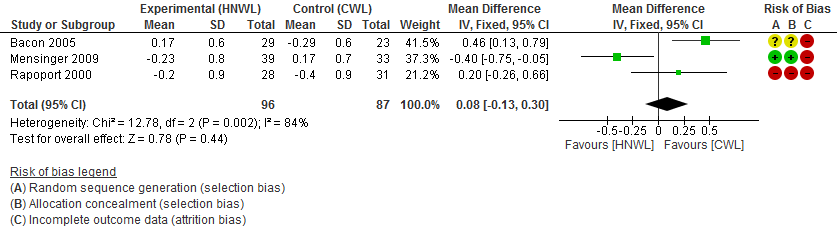

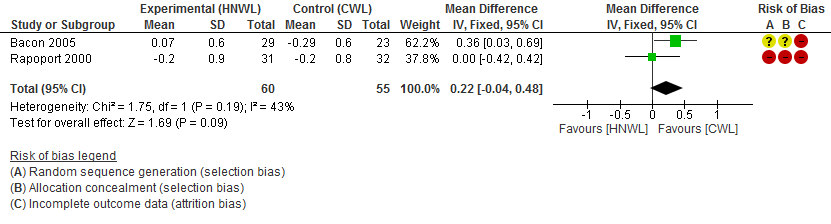


Figure 11. Meta-analysis for LDL at 40–52 weeks Figure 12. Meta-analysis for LDL at 53–104 weeks


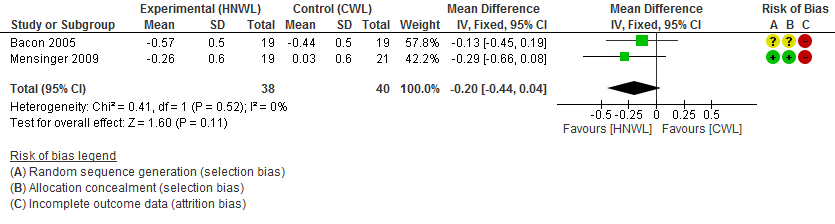

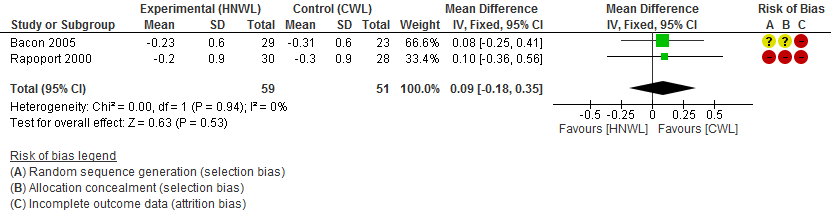


**Results for triglycerides**

The mean difference in change in triglyceride levels was in favour of CWL compared to HNWL programmes between weeks 8 and 19 (-0.12 mmoI/L[-0.39 to 0.16 ]) ([Figure](file:///C:\Users\ab5042\AppData\Local\Microsoft\Windows\Temporary%20Internet%20Files\Content.Outlook\L63T0RLL\004.01) 13) and weeks 20 to 39,( -0.17 mmoI/L[-0.42 to 0.08 ]) ( [Figure 14](file:///C:\Users\ab5042\AppData\Local\Microsoft\Windows\Temporary%20Internet%20Files\Content.Outlook\L63T0RLL\004.02)). Change in triglyceride level was in favour of the HNWL programmes (-0.04 mmoI/L[-0.35 to 0.26]) at weeks 40 to 52 (Figure 15). The single study ([Mensinger 2009](file:///C:\\Users\\ab5042\\AppData\\Local\\Microsoft\\Windows\\Temporary%20Internet%20Files\\Content.Outlook\\L63T0RLL\\Mensinger%202009)) provided data from weeks 53 to 104 showed a greater improvement (-0.16 mmoI/L[-0.64 to 0.32]) in triglyceride levels in the CWL compared to the HNWL programme (Figure 16).

Figure 13. Meta-analysis for Triglycerides at 8–19 weeks Figure 14. Meta-analysis for Triglycerides at 20–39 weeks


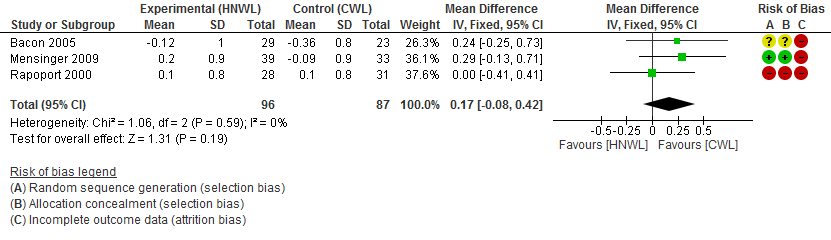

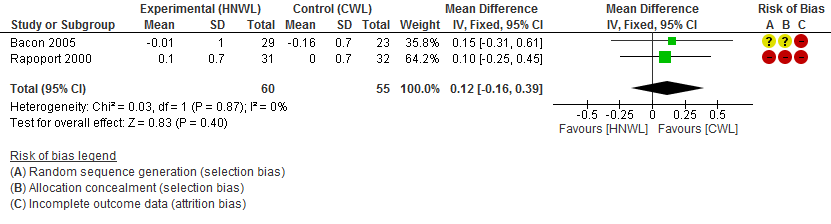


Figure 15. Meta-analysis for Triglycerides at 40–52 weeks Figure 16. Meta-analysis for Triglycerides at 53–104 weeks


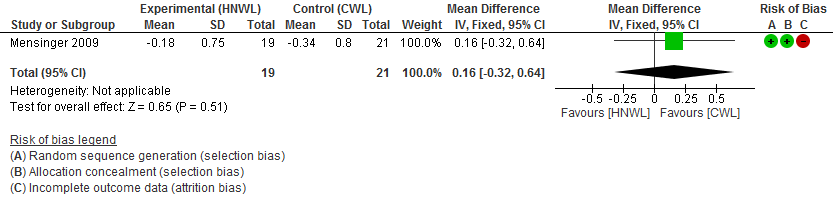

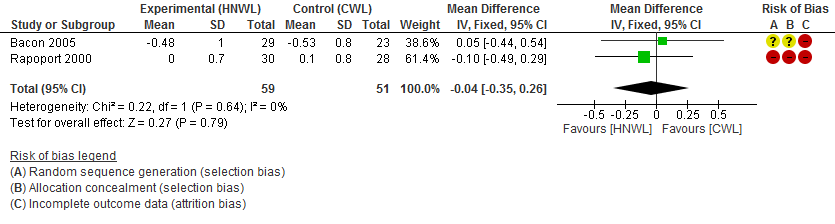


**Results for total Cholesterol-HDL Ratio**

Figure 17. Meta-analysis for Total cholesterol-HDL at 8–19 weeks Figure 18. Meta-analysis for Total cholesterol-HDL, 20–39 weeks


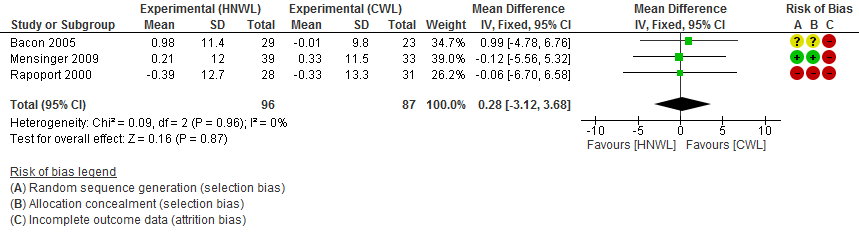

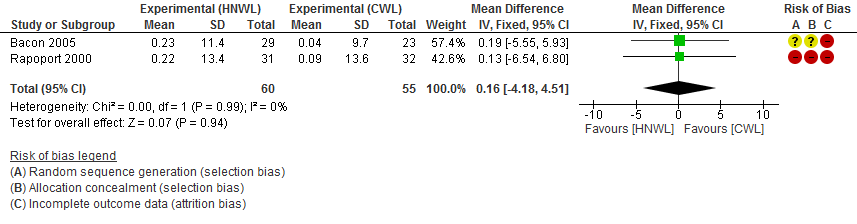


Figure 19. Meta-analysis for Total cholesterol-HDL at 40–52 weeks Figure 20. Meta-analysis for Total cholesterol-HDL, 53–104weeks


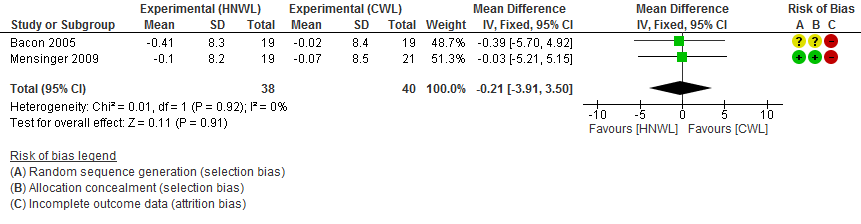

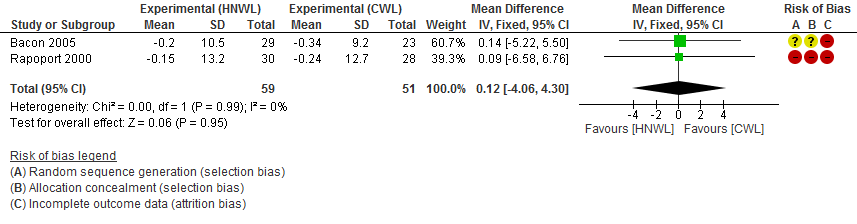


**Additional meta-analysis for blood Pressure**

Figure 21. Meta-analysis for Systolic BP at 8–19 weeks Figure 22. Meta-analysis for Systolic BP at 20–39 weeks


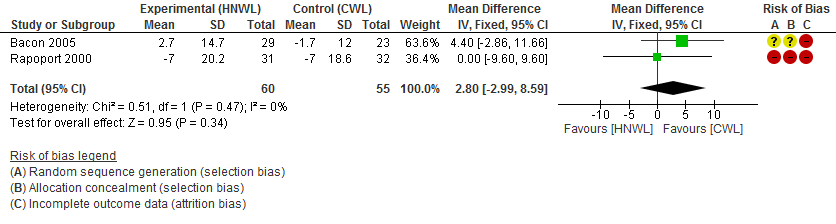

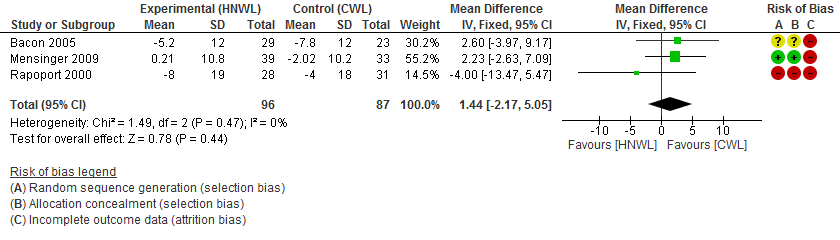


Figure 23. Meta-analysis for Systolic BP at 40–52 weeks


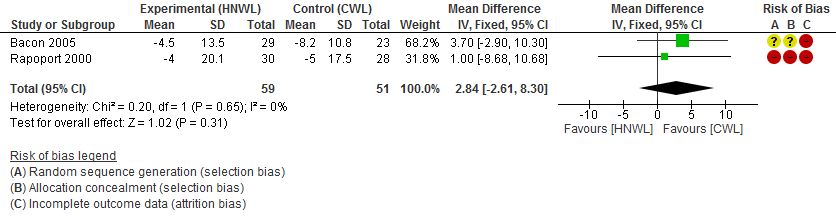


Figure 24. Meta-analysis for Systolic BP at 53–104 weeks


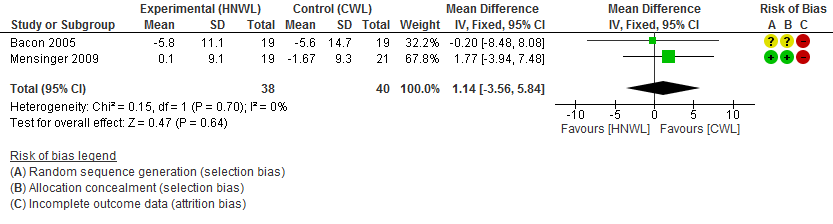


Figure 25. Meta-analysis for Diastolic BP at 8–19 weeks


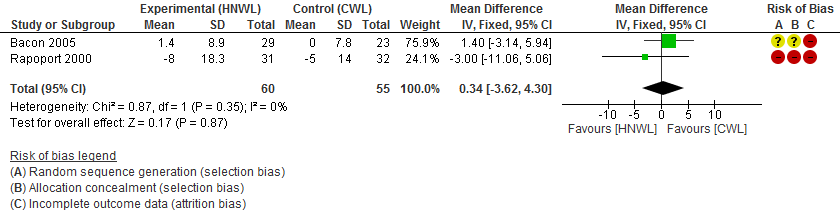


Figure 26. Meta-analysis for Diastolic BP at 20–39 weeks


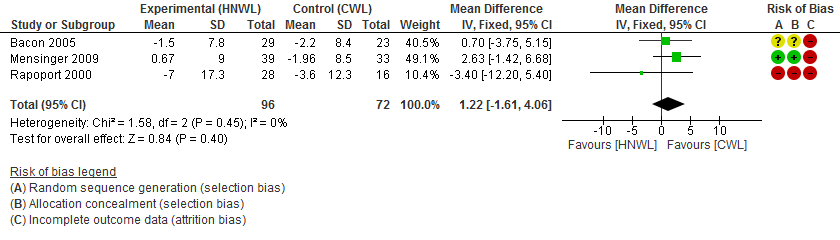


Figure 27. Meta-analysis for Diastolic BP at 40–52 weeks


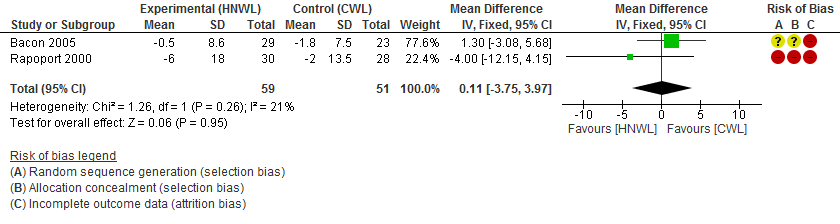


Figure 28. Meta-analysis for Diastolic BP at 53–104 weeks


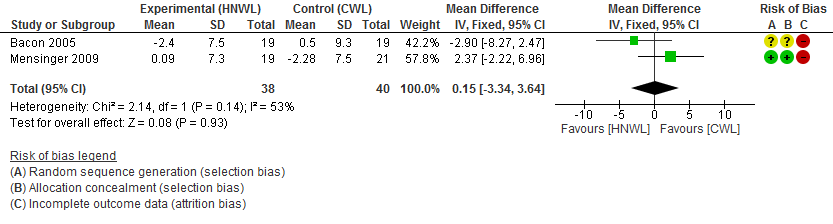


**Additional meta-analyses for weight**

Figure 29. Meta-analysis for Weight at 8–19 weeks
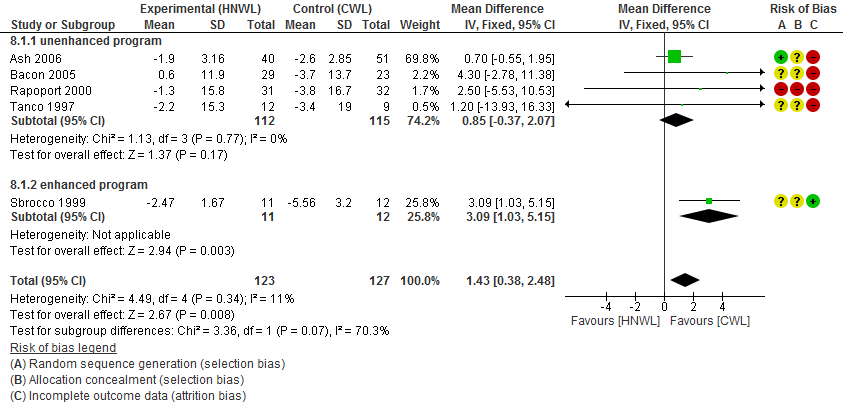


Figure 30. Meta-analysis for Weight at 8–19 weeks (excluding studies with high bias)


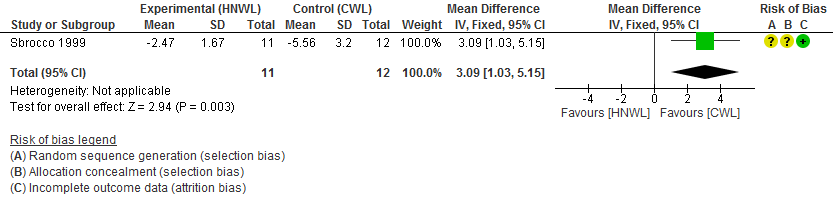


Figure 31. Meta-analysis for Weight at 20–39 weeks


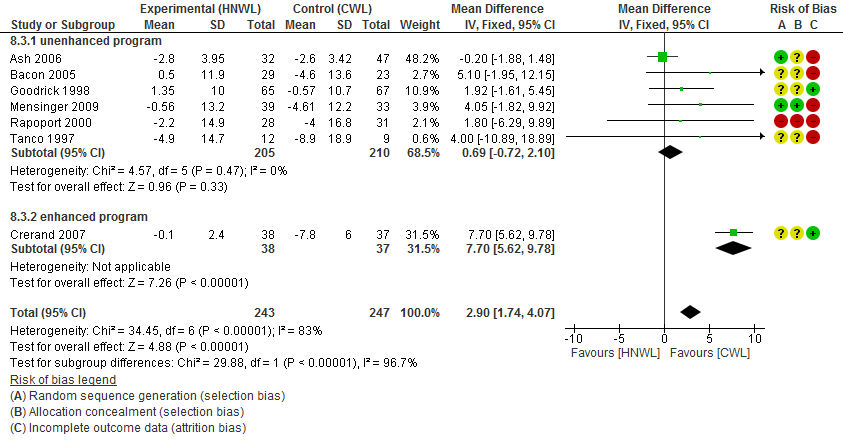


Figure 32. Meta-analysis for Weight at 20–39 weeks (excluding studies with high bias)


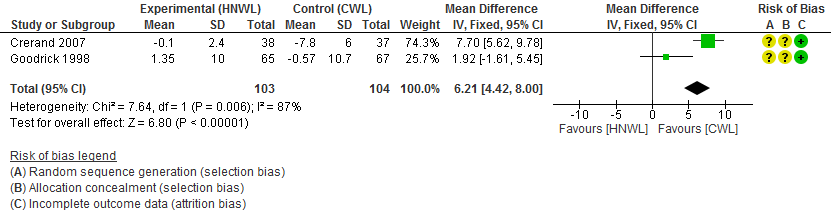


Figure 33. Meta-analysis for Weight at 40–52 weeks


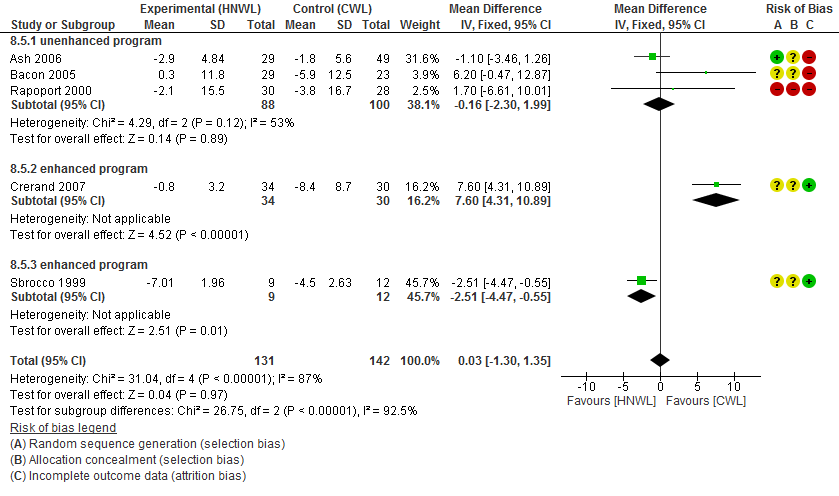


Figure 34 Meta-analysis for Weight at 40–52 weeks (excluding studies with high bias)


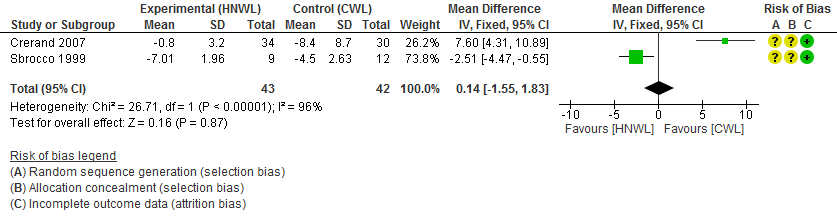


Additional meta-analyses for Secondary Outcomes

Figure 35. Meta-analysis for Daily energy expenditure at 8–19 weeks


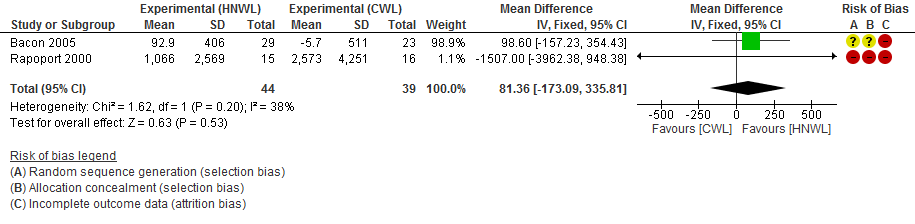


Figure 36. Meta-analysis for Daily energy expenditure at 20–39 weeks


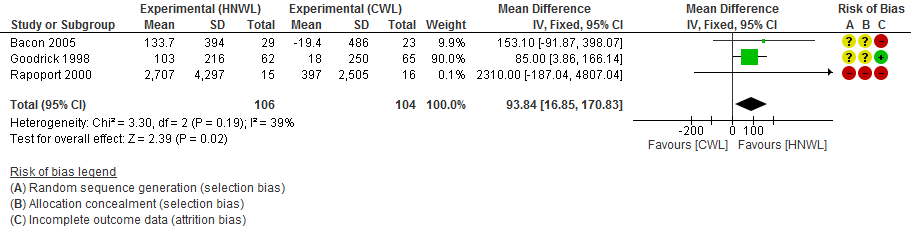


Figure 37. Meta-analysis for Daily energy expenditure at 40–52 weeks


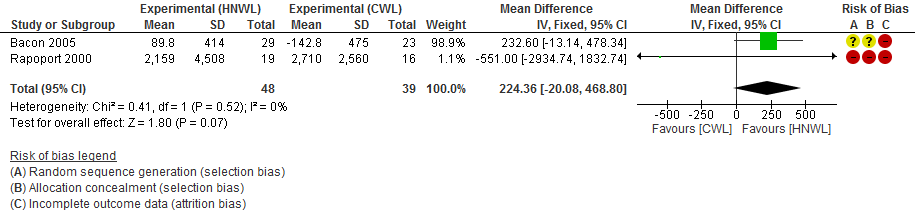


Figure 38. Meta-analysis for Daily energy expenditure at 53–104 weeks


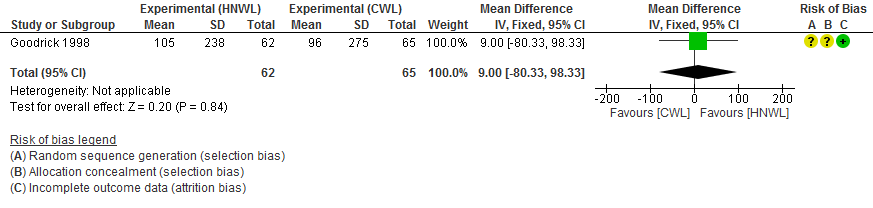


Figure 40. Meta-analysis for Self-esteem at 8–19 weeks


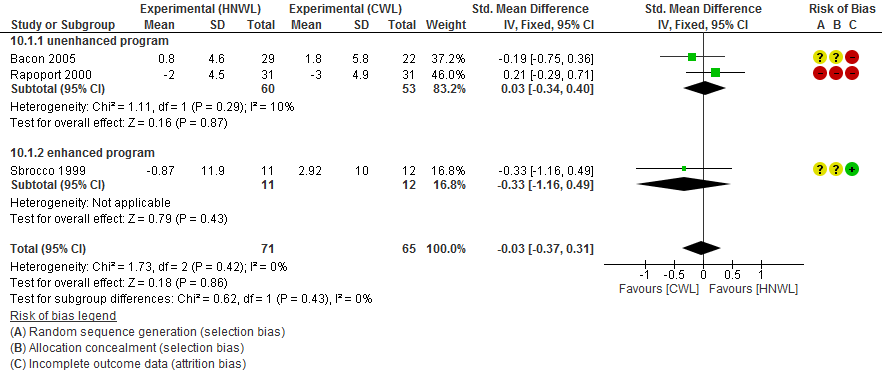


Figure 41. Meta-analysis for Self-esteem at 20–39 weeks


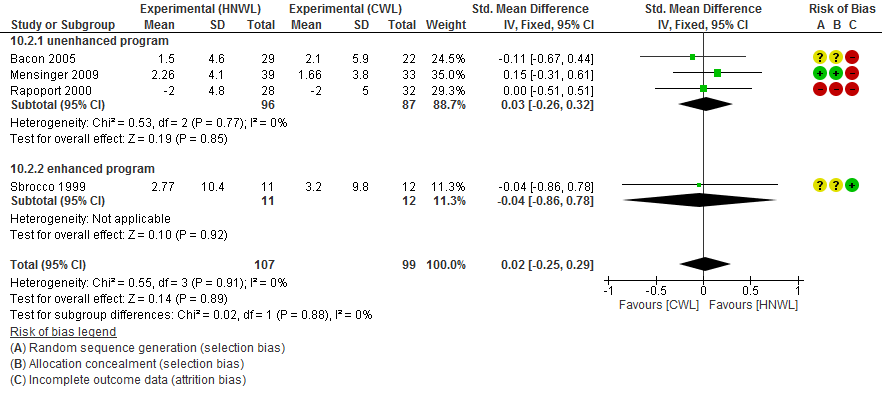


Figure 42. Meta-analysis for Self-esteem at 40–52 weeks


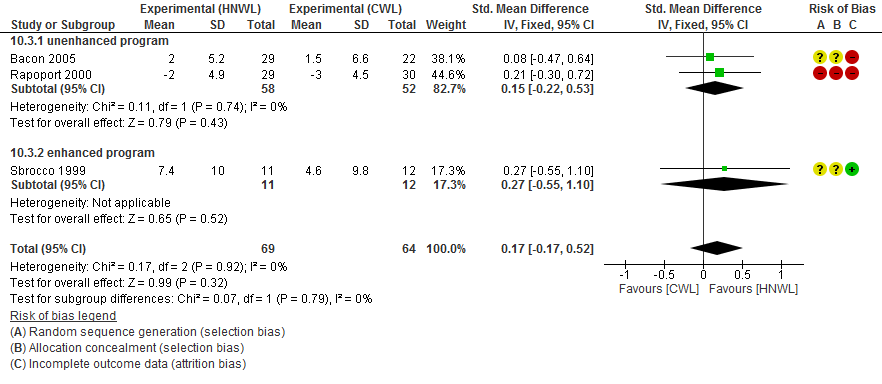


Figure 43. Meta-analysis for Self-esteem at 53–104 weeks


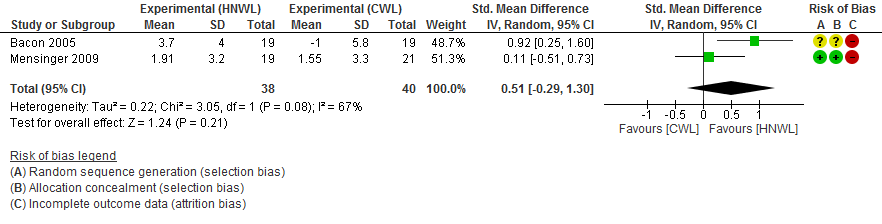


Figure 44. **Meta-analysis for Body Image Avoidance at 8–19 weeks**


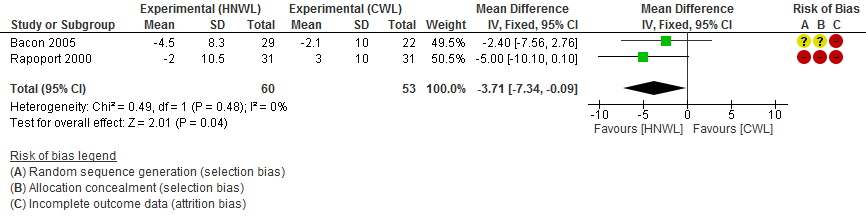


Figure 45. Meta-analysis for Body Image Avoidance at 20–39 weeks


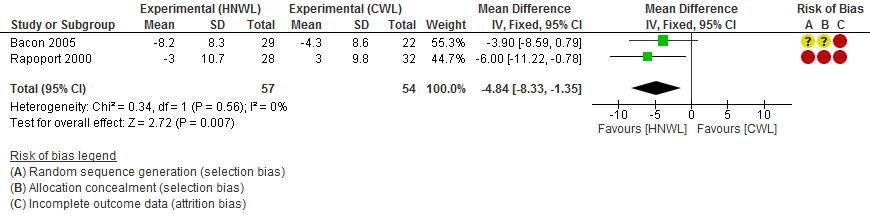


Figure 46. Meta-analysis for Body Image Avoidance at 40–52 weeks


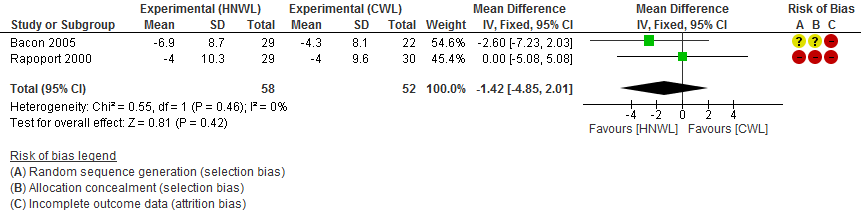


Figure 47. Meta-analysis for Depression at 8–19 weeks


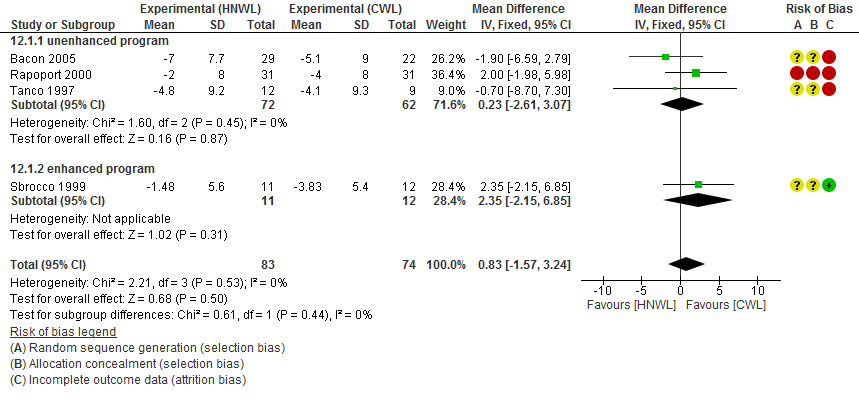


Figure 48. Meta-analysis for Depression at 20–39 weeks


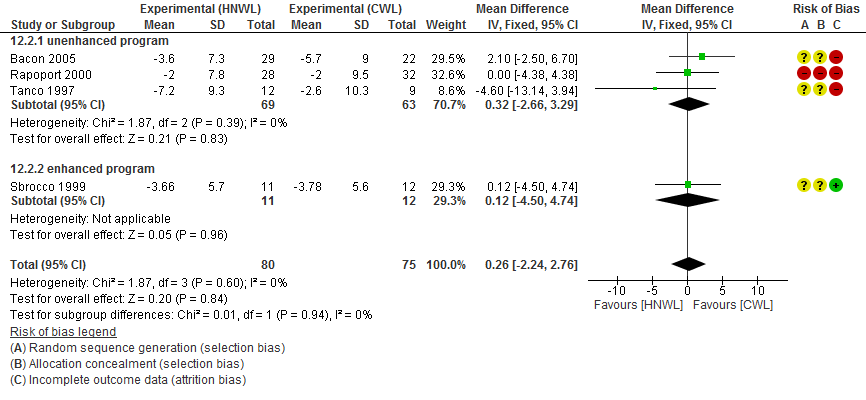


Figure 49. Meta-analysis for Depression at 40–52 weeks


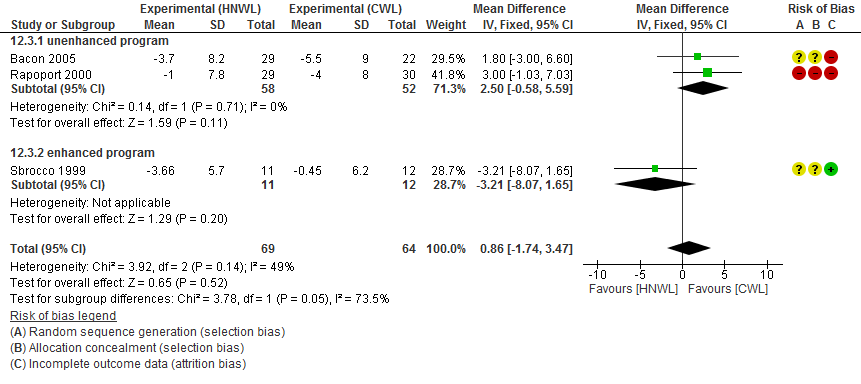


Figure 50. Meta-analysis for Binge eating at 8–19 weeks


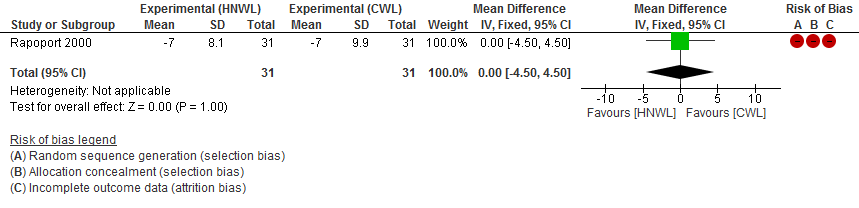


Figure 51. Meta-analysis for Binge eating at 20–39 weeks


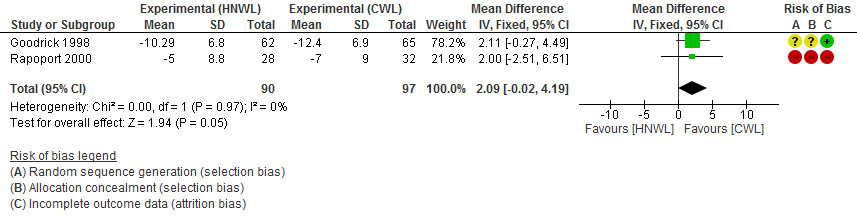


Figure 52. Meta-analysis for Drive for thinness at 8–19 weeks


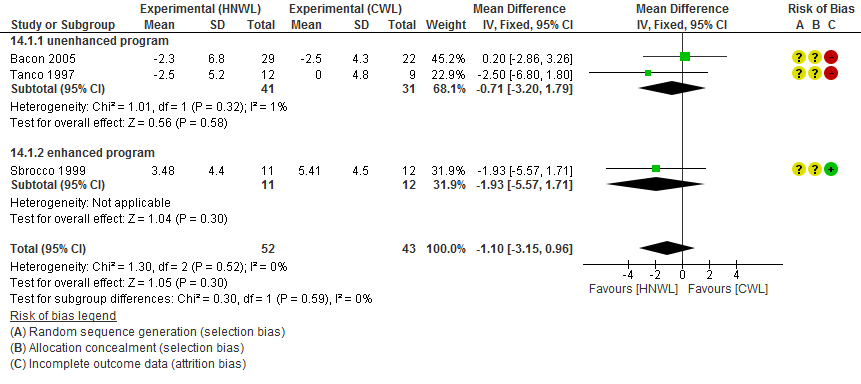


Figure 53. Meta-analysis for Drive for thinness at 20–39 weeks


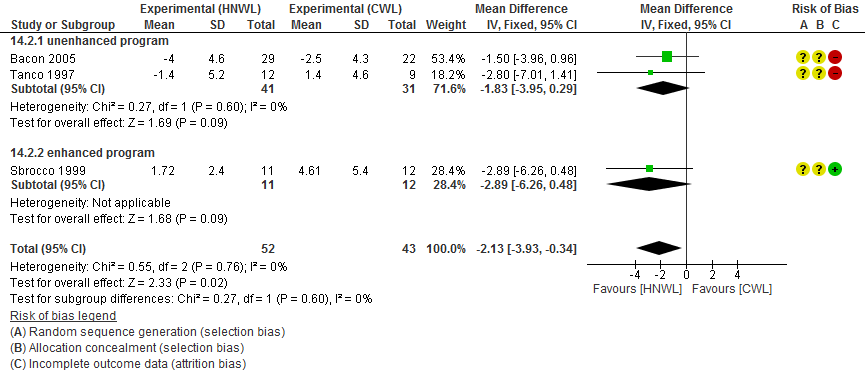


Figure 54. Meta-analysis of the Results Drive for thinness at 40–52 weeks


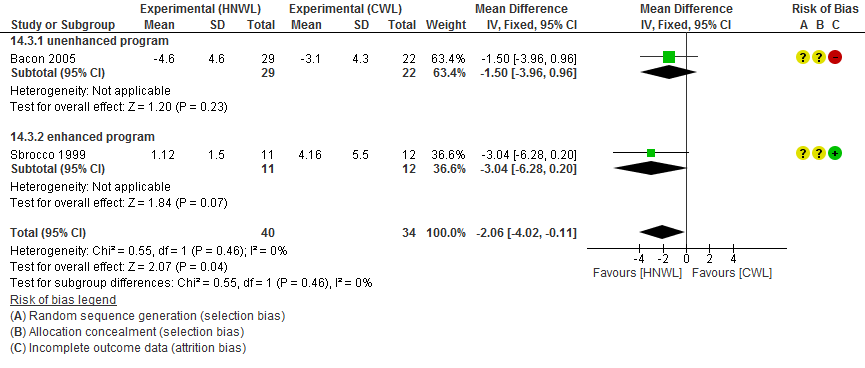


Figure 55. Meta-analysis for Bulimia at 8–19 weeks


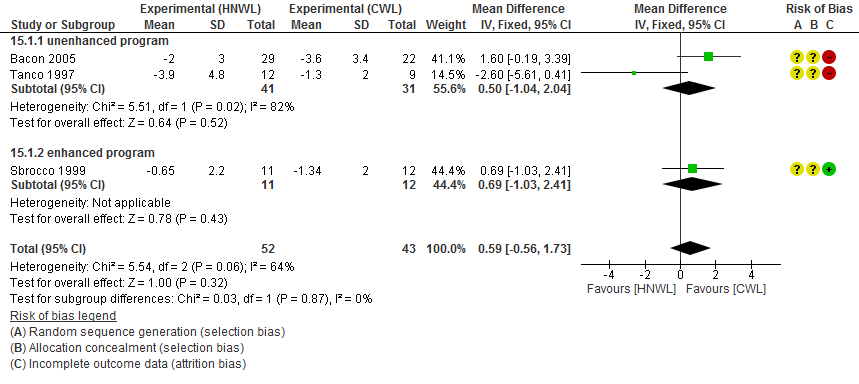


Figure 56. Meta-analysis for Bulimia at 20–39 weeks


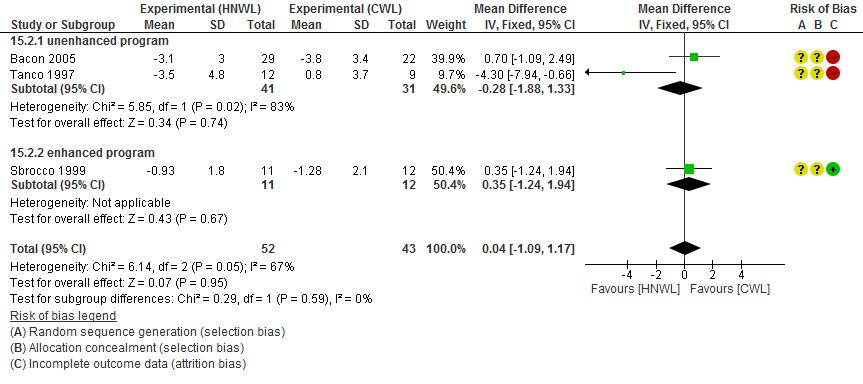


Figure 57. Meta-analysis for Bulimia at 40–52 weeks


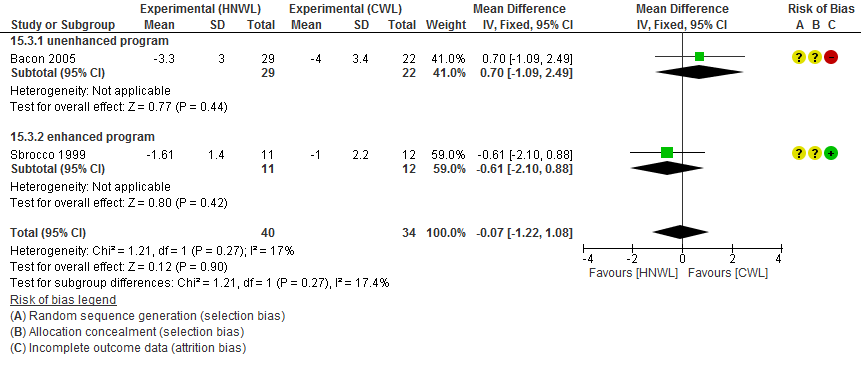


Figure 58. Meta-analysis for Body dissatisfaction at 8–19 weeks


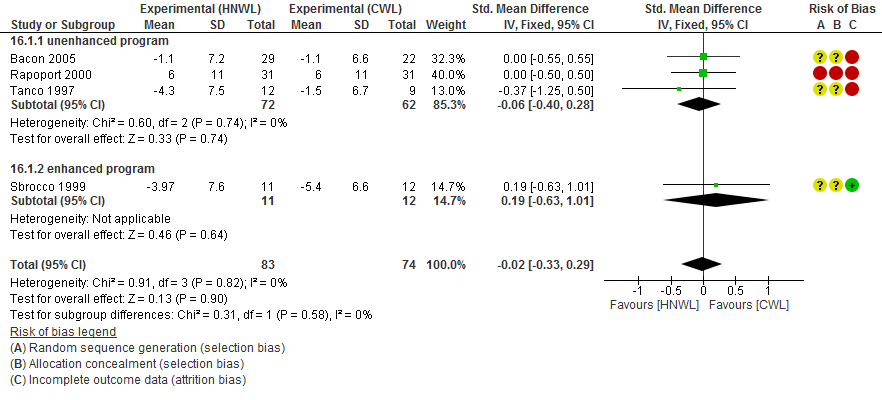


Figure 59. Meta-analysis for Body dissatisfaction at 20–39 weeks


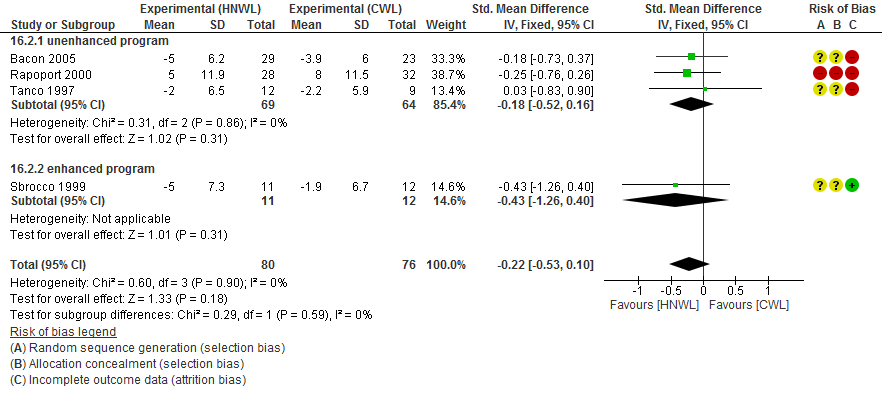


Figure 60. Meta-analysis for Body dissatisfaction at 40–52 weeks


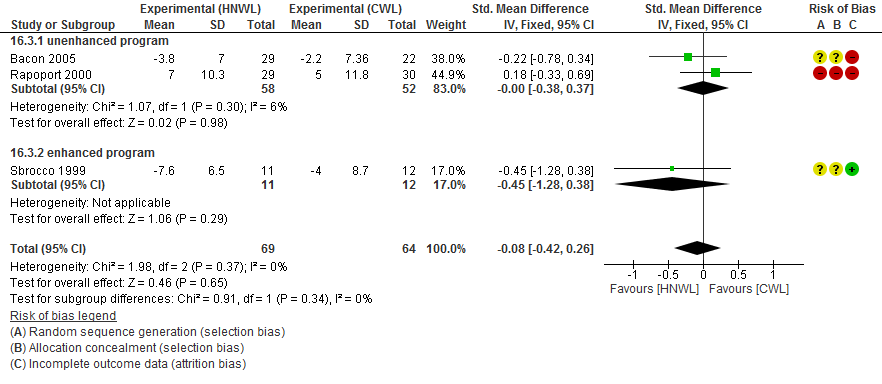


Figure 61. Meta-analysis for Hunger at 8–19 weeks


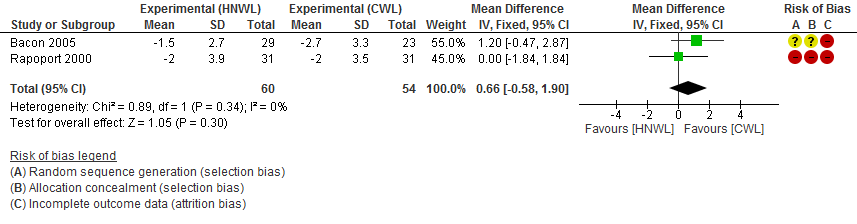


Figure 62. Meta-analysis for Hunger at 20–39 weeks


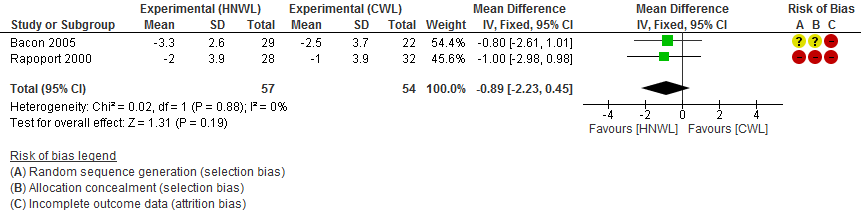


Figure 63. Meta-analysis for Hunger at 40–52 weeks


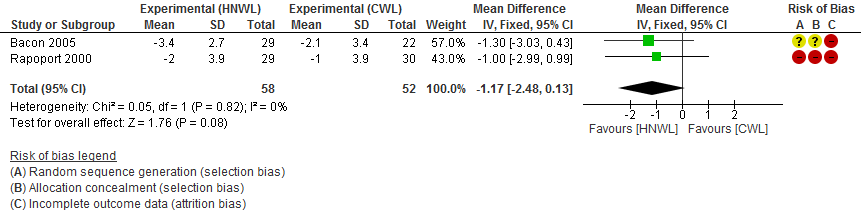


Figure 64. Meta-analysis for Disinhibition at 8–19 weeks


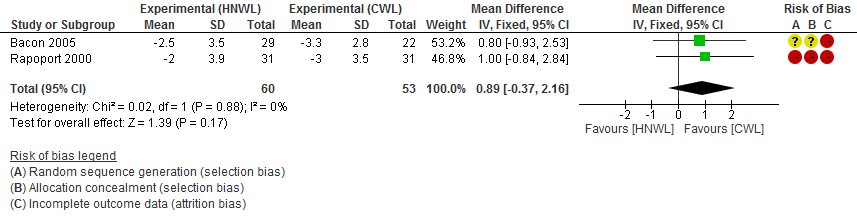


Figure 65. Meta-analysis for Disinhibition at 20–39 weeks


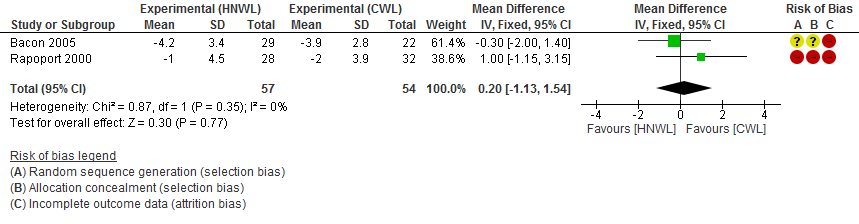


Figure 66. Meta-analysis for Disinhibition at 40–52 weeks


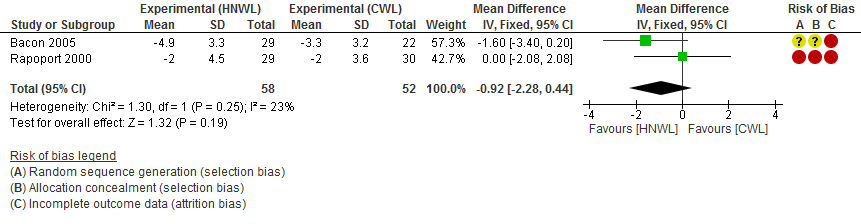


Figure 67. Meta-analysis for Restrained eating behaviour at 8–19 weeks


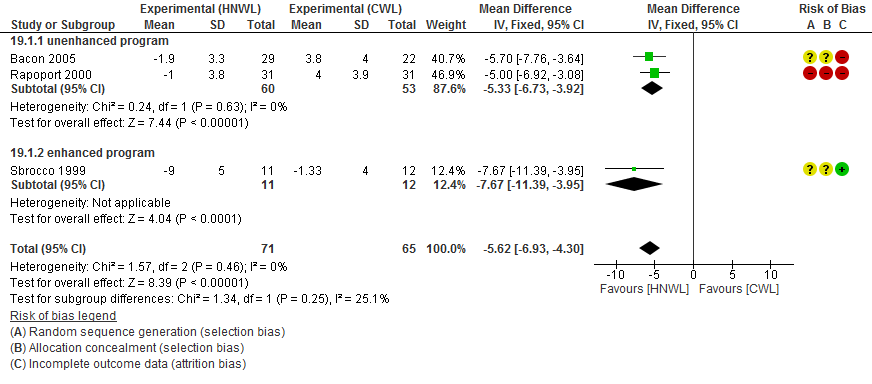


Figure 68. Meta-analysis for Restrained eating behaviour at 20–39 weeks


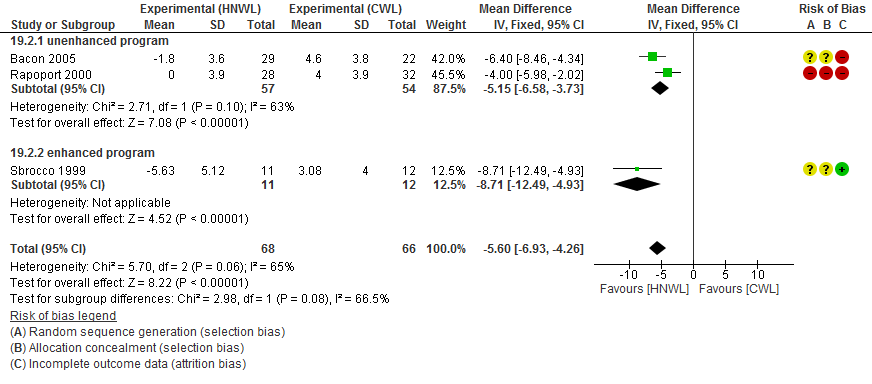


Figure 69. Meta-analysis for Restrained eating behaviour at 40–52 weeks


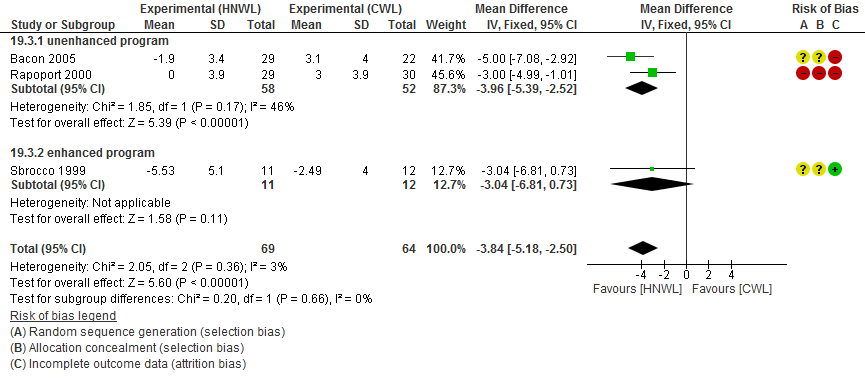


Table 1. [Characteristics of excluded studies](file:///E:\Tez%20writing\SR%20results\CHARACTERISTICS_OF_EXCLUDED_STUDIES)

| **Excluded studies** | **Reason for exclusion** |
| --- | --- |
| Alberts, Thewissen, & Raes, 2012 | Control group was waiting list group, not CWL. |
| Anglin, 2012 | Duration of study was 6 weeks. |
| Attux et a.l, 2011 | Not a randomised control trial, no weight loss control group. Participants with a BMI< 25 kg/m^2^ were not excluded. |
| Augustus-Horvath & Tylka, 2011 | Participants with a BMI< 25 kg/m^2^ were not excluded. No weight loss control group. |
| Blevins, 2008 | Intervention was promoting calorie restricted diet, not HNWL philosophy. |
| Bolton et al., 2010 | Not a randomised controlled trial. |
| Bradshaw, Horwath, Katzer, & Gray, 2009 | No weight loss control group, both groups are HNWL philosophy. |
| Burnette & Finkel, 2012 | Intervention was LEARN program that promoted weight loss, not HNWL philosophy. |
| Carels et al., 2013 | Intervention was promoting weight loss, not HNWL philosophy. |
| Carrier, Steinhardt, & Bowman, 1994 | Not a randomised controlled study (Pre and post survey study). |
| Carroll, Borkoles, & Polman, 2007 | Control group was delayed start, not CWL. |
| Carroll, Marshall, Ingle, & Borkoles, 2012 | Intervention was promoting calorie restricted diet, not HNWL philosophy. Control group was delayed start, not CWL. |
| Ciliska, 1998 | No weight loss control group, both groups are HNWL philosophy |
| Cole, 2006 | Participants with a BMI< 25 kg/m^2^ were not excluded. Intervention was promoting weight loss, not HNWL philosophy. |
| Cole & Horacek, 2010 | Participants with a BMI< 25 kg/m^2^ were not excluded. Intervention was promoting weight loss, not HNWL philosophy. This study is referenced in other published articles that were excluded from the research because the study was duplicated ([Cole and Horacek, 2009](file:///E:\Tez%20writing\SR%20results\Cole%202009)). |
| Cooper & Fairburn, 2001 | Not a randomised controlled trial. |
| Dale et al., 2009 | Intervention was promoting weight loss, not HNWL philosophy. |
| Daumit et a.l, 2013 | \| Intervention was promoting calorie restricted diet, not HNWL philosophy. \| \| --- \| |
| Dodd et al, 2014 | Control group was an intervention for preventing weight gain in pregnant women i.e. not CWL. |
| Forman, Butryn, Hoffman, & Herbert, 2009 | Intervention was LEARN program that promoted weight loss, not HNWL philosophy. |
| Fuller, 1994 | Intervention was promoting weight loss, not HNWL philosophy. |
| Glenn, 2003 | No randomised control trial study. |
| Gravel et al., 2014 | Participants with a BMI< 25 kg/m^2^ were not excluded. Control group was waiting list control group, not CWL. |
| Green, Elliman, & Kretsch, 2005 | Intervention was promoting calorie restricted diet, not HNWL philosophy. |
| Hawley et al., 2008 | No weight loss control group, all 3 groups are HNWL philosophy. This study is referenced in other published articles that were excluded from the research because the study was duplicated ([Katzer et al., 2008](file:///E:\\Tez%20writing\\SR%20results\\Katzer%202008)). |
| Heymsfield et al., 2010 | Intervention was not HNWL philosophy. |
| Hsu, Buckworth, Focht, & O'Connell, 2013 | Control group was exercising only intervention (EX), not CWL. |
| Ikeda et al., 2005 | Not a randomised controlled trial. |
| Jefferey et al., 1993 | Intervention was promoting weight loss, not HNWL philosophy. |
| Jefferson, 2005 | Intervention was promoting weight loss, not HNWL philosophy. |
| Reel & Stuart,, 2012 | Not a randomised controlled trial. |
| Khazaal et al., 2007 | Participants with a BMI< 25 kg/m^2^ were not excluded. Intervention was not HNWL philosophy. |
| Kim, Lee, Ahn, & Lee, 2010 | Participants with a BMI< 25 kg/m^2^ were not excluded. No weight loss control group. |
| Leblanc et al,. 2012 | Intervention was promoting HNWL philosophy. Control group was delayed start, not CWL. This study is referenced in other four published articles that were excluded from the research because they were duplicated ([Cote et al., 2011](file:///E:\Tez%20writing\SR%20results\Cote%202011); [Gagnon-Girouard et al., 2010](file:///E:\Tez%20writing\SR%20results\Gagnon-Girouard%202010); [Provencher et al., 2007](file:///E:\Tez%20writing\SR%20results\Provencher%202007); [Provencher et al., 2009](file:///E:\Tez%20writing\SR%20results\Provencher%202009)). |
| Lillis, Hayes, Bunting, & Masuda, 2009 | Patients had to lose weight before start of study and control group was waiting list group, not CWL. |
| Lombard, Deeks, Jolley, Ball, & Teede, 2010 | Participants with a BMI< 25 kg/m^2^ were not excluded. No weight loss control group, not CWL. Aim of the intervention was to prevent weight gain. |
| McReynolds, Lutz, Paulsen, & Kohrs, 1976 | Intervention group was not HNWL philosophy, control group was delayed start not CWL. |
| Miller, Kristeller, Headings, & Nagaraja, 2013 | Intervention was promoting weight loss, not HNWL philosophy. |
| Munro, 2002 | Intervention is not HNWL, but a brief therapy to promote weight loss at a time of likely relapse. |
| Murawski, 2007 | No weight loss control group. |
| Perri et al., 1988 | Intervention was promoting weight loss, not HNWL philosophy. |
| Perri et al., 2001 | Intervention was promoting weight loss, not HNWL philosophy. |
| Beeken et al., 2012 | Intervention was promoting weight loss, not HNWL philosophy. |
| Rickel, 2008 | Intervention was low-calorie diet to promote weight loss, not HNWL philosophy. |
| Riebe et al., 2005 | Not a randomised controlled trial. |
| Rippe et al., 1998 | Intervention was promoting weight loss, not HNWL philosophy. |
| Robison, Putnam and McKibbin, 2007 | Not a randomised controlled trial. |
| Rotatori, Switzky, & Fox, 1981 | No HNWL intervention group, both groups are based on CWL philosophy. |
| Shaw, O'Rourke, Del, & Kenardy, (2005) | Systematic review. |
| Smith, 2007 | Control group was waiting list control group, not CWL. |
| Steinhardt, Bezner &Adams, 1999 | Not a randomised controlled study (Quasi-experimental study). |
| Stuart, 1971 | Intervention was not HNWL philosophy. |
| Tapper et al., 2009 | Mindfulness intervention was designed for promoting weight loss, not HNWL philosophy. |
| TerBogt et al., 2011 | Intervention was promoting 5–10% weight loss, not HNWL philosophy. |
| Toft et al., 2008 | Participants with a BMI< 25 kg/m^2^ were not excluded. Intervention was not HNWL philosophy. |
| Turner et al., 2008 | Participants with a BMI< 25 kg/m^2^ were not excluded. No HNWL intervention group, all 3 groups are based on CWL philosophy. |
| Vinkers et al., 2014 | Intervention was promoting weight loss, not HNWL philosophy |
| Von Gruenigen et al., 2012 | Intervention was promoting weight loss, not HNWL philosophy. |

**Excluded studies**

Alberts, H., Thewissen, R., & Raes, L. (2012). Dealing with problematic eating behaviour. The effects of a mindfulness-based intervention on eating behaviour, food cravings, dichotomous thinking and body image concern. *Appetite*, *58*(3), 847-851. http://dx.doi.org/10.1016/j.appet.2012.01.009

Anglin, J. (2012). Assessing the effectiveness of intuitive eating for weight loss - pilot study. *Nutrition And Health*, *21*(2), 107-115. http://dx.doi.org/10.1177/0260106012459994

Attux, C., Martini, LC., Araujo, CM., Roma, AM., Reis, AF., & Bressan, RA. (2011). The effectiveness of a non-pharmacological intervention for weight gain management in severe mental disorders: results from a national multicentric study. *Revista Brasileira de Psiquiatria*, 33(2), 117-121.

Augustus-Horvath, C. & Tylka, T. (2011). The acceptance model of intuitive eating: A comparison of women in emerging adulthood, early adulthood, and middle adulthood. *Journal Of Counseling Psychology*, *58*(1), 110-125. http://dx.doi.org/10.1037/a0022129

Blevins, NC. (2008). Mindfulness meditation as an intervention for body image and weight management in college women: A pilot study. *Florida, USA: University of Florida*.

Bolton, K., Swinburn, B., Kremer, P., Waters, E., Gibbs, L., Haby, M, et al. (2010). Evaluation design of complex community-based interventions: the "Go for your life" Health Promoting Communities: Being Active and Eating Well initiative. *In: Obesity Reviews*. Vol. 11. Conference Publication: 11th International Congress on Obesity, ICO 2010 Stockholm Sweden Conference, 2010:455-56.

Bradshaw, A., Horwath, C., Katzer, L., & Gray, A. (2009). Non-dieting group interventions for overweight and obese women: what predicts non-completion and does completion improve outcomes?. *Public Health Nutr.*, *13*(10), 1622-1628. <http://dx.doi.org/10.1017/s1368980009992977>

Burnette, J. & Finkel, E. (2012). Buffering against weight gain following dieting setbacks: An implicit theory intervention. *Journal Of Experimental Social Psychology*, *48*(3), 721-725. <http://dx.doi.org/10.1016/j.jesp.2011.12.020>

Carels, R., Burmeister, J., Koball, A., Oehlhof, M., Hinman, N., & LeRoy, M. et al. (2013). A randomized trial comparing two approaches to weight loss: Differences in weight loss maintenance. *Journal Of Health Psychology*, *19*(2), 296-311. http://dx.doi.org/10.1177/1359105312470156

Carrier, K., Steinhardt, M., & Bowman, S. (1994). Rethinking Traditional Weight Management Programs: A 3-Year Follow-Up Evaluation of a New Approach. *The Journal Of Psychology*, *128*(5), 517-535. http://dx.doi.org/10.1080/00223980.1994.9914910

Carroll, S., Borkoles, E., & Polman, R. (2007). Short-term effects of a non-dieting lifestyle intervention program on weight management, fitness, metabolic risk, and psychological well-being in obese premenopausal females with the metabolic syndrome. *Appl. Physiol. Nutr. Metab.*, *32*(1), 125-142. <http://dx.doi.org/10.1139/h06-093>

Carroll, S., Marshall, P., Ingle, L., & Borkoles, E. (2012). Cardiorespiratory fitness and heart rate recovery in obese premenopausal women. *Scandinavian Journal Of Medicine & Science In Sports*, *22*(6), e133-e139. <http://dx.doi.org/10.1111/j.1600-0838.2012.01522.x>

Ciliska, D. (1998). Evaluation of Two Nondieting Interventions for Obese Women. *Western Journal Of Nursing Research*, *20*(1), 119-135. <http://dx.doi.org/10.1177/019394599802000108>

Cole, RE. (2006) Intuitive eating non-dieting approach to weight management: Pilot program for fort drum dod beneficiaries. *Syracuse, USA: Syracuse University*.

Cole, R. and Horacek, T. (2010). Effectiveness of the My Body Knows When Intuitive-eating Pilot Program. *am j hlth behav*, 34(3), pp.286-297.

Cooper, Z. & Fairburn, C. (2001). A new cognitive behavioural approach to the treatment of obesity. *Behaviour Research And Therapy*, *39*(5), 499-511. <http://dx.doi.org/10.1016/s0005-7967(00)00065-6>

Dale, K., McAuley, K., Taylor, R., Williams, S., Farmer, V., & Hansen, P. et al. (2009). Determining optimal approaches for weight maintenance: a randomized controlled trial. *Canadian Medical Association Journal*, *180*(10), E39-E46. http://dx.doi.org/10.1503/cmaj.080974

Daumit, G., Dickerson, F., Wang, N., Dalcin, A., Jerome, G., & Anderson, C. et al. (2013). A Behavioral Weight-Loss Intervention in Persons with Serious Mental Illness. *New England Journal Of Medicine*, *368*(17), 1594-1602. <http://dx.doi.org/10.1056/nejmoa1214530>

Dodd, J., Turnbull, D., McPhee, A., Deussen, A., Grivell, R., & Yelland, L. et al. (2014). Antenatal lifestyle advice for women who are overweight or obese: LIMIT randomised trial. *BMJ*, *348*(feb10 3), g1285-g1285. <http://dx.doi.org/10.1136/bmj.g1285>

Forman, E., Butryn, M., Hoffman, K., & Herbert, J. (2009). An Open Trial of an Acceptance-Based Behavioral Intervention for Weight Loss. *Cognitive And Behavioral Practice*, *16*(2), 223-235. http://dx.doi.org/10.1016/j.cbpra.2008.09.005

Fuller, PR. (1994). Improving the behavioral treatment of obesity in adults. *Florida, USA: University of Florida.*

Glenn, AG. (2003). Is It Necessary to be Thin to beHealthy? *Harvard Health Policy Review*, 4(2),40-47.

Gravel, K., Deslauriers, A., Watiez, M., Dumont, M., Dufour Bouchard, A., & Provencher, V. (2014). Sensory-Based Nutrition Pilot Intervention for Women. *Journal Of The Academy Of Nutrition And Dietetics*, *114*(1), 99-106. http://dx.doi.org/10.1016/j.jand.2013.06.354

Green, M., Elliman, N., & Kretsch, M. (2005). Weight loss strategies, stress, and cognitive function: Supervised versus unsupervised dieting. *Psychoneuroendocrinology*, *30*(9), 908-918. http://dx.doi.org/10.1016/j.psyneuen.2005.05.005

Hawley, G., Horwath, C., Gray, A., Bradshaw, A., Katzer, L., Joyce, J., & O'Brien, S. (2008). Sustainability of health and lifestyle improvements following a non-dieting randomised trial in overweight women. *Preventive Medicine*, *47*(6), 593-599. http://dx.doi.org/10.1016/j.ypmed.2008.08.008

Heymsfield, SB., Thomas, D., Martin, CK., Shen, W. &, Strauss, B, (2010). Bosy-Westphal A. Kinetics of fat-free mass relative to body weight loss with short-term diet and exercise treatments: Implications for new therapy safety evaluations. *Obesity Reviews*,11,178

Hsu, Y., Buckworth, J., Focht, B., & O'Connell, A. (2013). Feasibility of a Self-Determination Theory-based exercise intervention promoting Healthy at Every Size with sedentary overweight women: Project CHANGE. *Psychology Of Sport And Exercise*, *14*(2), 283-292. http://dx.doi.org/10.1016/j.psychsport.2012.11.007

Ikeda, J., Amy, NK., Ernsberger, P., Gaesser, GA., Berg, FM., Clark, CA., et al. (2005). The National Weight Control Registry: A critique. *Journal of Nutrition Education and Behavior* , 37(4), 203-205.

Jeffery, R., Forster, J., Baxter, J., French, S., & Kelder, S. (1993). An Empirical Evaluation of the Effectiveness of Tangible Incentives in Increasing Participation and Behavior Change in a Worksite Health Promotion Program. *American Journal Of Health Promotion*, *8*(2), 98-100. http://dx.doi.org/10.4278/0890-1171-8.2.98

Jefferson, ML. (2005). The effects of self-monitoring psychological states on behavioral weight management treatment*. Ohio, USA: Ohio State University.*

J Reel, J. & R Stuart, A. (2012). Is The “Health at Every Size” Approach Useful for Addressing Obesity?. *J Community Med Health Edu*, *02*(04). http://dx.doi.org/10.4172/jcmhe.1000e105

Khazaal, Y., Fresard, E., Rabia, S., Chatton, A., Rothen, S., & Pomini, V. et al. (2007). Cognitive behavioural therapy for weight gain associated with antipsychotic drugs. *Schizophrenia Research*, *91*(1-3), 169-177. http://dx.doi.org/10.1016/j.schres.2006.12.025

Kim, M., Lee, S., Ahn, Y., & Lee, H. (2010). Lifestyle advice for Korean Americans and native Koreans with hypertension. *Journal Of Advanced Nursing*, *67*(3), 531-539. http://dx.doi.org/10.1111/j.1365-2648.2010.05504.x

Leblanc, V., Provencher, V., Bégin, C., Corneau, L., Tremblay, A., & Lemieux, S. (2012). Impact of a Health-At-Every-Size intervention on changes in dietary intakes and eating patterns in premenopausal overweight women: Results of a randomized trial. *Clinical Nutrition*, *31*(4), 481-488. http://dx.doi.org/10.1016/j.clnu.2011.12.013

Lillis, J., Hayes, S., Bunting, K., & Masuda, A. (2009). Teaching Acceptance and Mindfulness to Improve the Lives of the Obese: A Preliminary Test of a Theoretical Model. *Annals Of Behavioral Medicine*, *37*(1), 58-69. http://dx.doi.org/10.1007/s12160-009-9083-x

Lombard, C., Deeks, A., Jolley, D., Ball, K., & Teede, H. (2010). A low intensity, community based lifestyle programme to prevent weight gain in women with young children: cluster randomised controlled trial. *BMJ*, *341*(jul13 1), c3215-c3215. http://dx.doi.org/10.1136/bmj.c3215

McReynolds, W., Lutz, R., Paulsen, B., & Kohrs, M. (1976). Weight loss resulting from two behavior modification procedures with nutritionists as therapists. *Behavior Therapy*, *7*(3), 283-291. http://dx.doi.org/10.1016/s0005-7894(76)80054-8

Miller, C., Kristeller, J., Headings, A., & Nagaraja, H. (2013). Comparison of a Mindful Eating Intervention to a Diabetes Self-Management Intervention Among Adults With Type 2 Diabetes: A Randomized Controlled Trial. *Health Education & Behavior*, *41*(2), 145-154. http://dx.doi.org/10.1177/1090198113493092

Munro, JF. (2002). Second-Order Change through Brief Therapy among Obese Clients of a University-Based Weight Management Program. University of Missouri, St. Louis*. Abstract in UMI:ProQuest Information and Learning Company*, Dissertation.

Murawski, ME. (2007). Problem solving and the management of obesity in women from underserved rural settings. *Florida, USA: University of Florida.*

Perri, M., McAllister, D., Gange, J., Jordan, R. and et al, (1988). Effects of four maintenance programs on the long-term management of obesity. *Journal of Consulting and Clinical Psychology*, 56(4), pp.529-534.

Perri, M., Nezu, A., McKelvey, W., Shermer, R., Renjilian, D. and Viegener, B. (2001). Relapse prevention training and problem-solving therapy in the long-term management of obesity. *Journal of Consulting and Clinical Psychology*, 69(4), pp.722-726.

Beeken, R., Croker, H., Morris, S., Leurent, B., Omar, R., Nazareth, I. and Wardle, J. (2012). Study protocol for the 10 Top Tips (10TT) Trial: Randomised controlled trial of habit-based advice for weight control in general practice. *BMC Public Health*, 12(1), p.667.

Rickel, KA. (20088). Response of African-American and Caucasian women in a rural setting to a lifestyle intervention for obesity. *Florida, USA: University of Florida.*

RIEBE, D., BLISSMER, B., GREENE, G., CALDWELL, M., RUGGIERO, L., STILLWELL, K. and NIGG, C. (2005). Long-term maintenance of exercise and healthy eating behaviors in overweight adults. *Preventive Medicine*, 40(6), pp.769-778.

Rippe, J., Price, J., Hess, S., Kline, G., DeMers, K., Damitz, S., Kreidieh, I. and Freedson, P. (1998). Improved Psychological Well-Being, Quality of Life, and Health Practices in Moderately Overweight Women Participating in a 12-Week Structured Weight Loss Program. *Obesity Research*, 6(3), pp.208-218.

Robison, J., Putnam, K. and McKibbin, L. (2007). Health at Every Size: A Compassionate, Effective Approach for Helping Individuals with Weight-Related Concerns--Part I. *Workplace Health & Safety*, 55(4), pp.143-150.

Rotatori ,AF., Switzky, HN., & Fox, R. (1981). Behavioral Weight Reduction Procedures forObese Mentally Retarded Individuals: A Review. *American Association on Mental Deficiency*, 19(4), 157-216.

Shaw, K., O'Rourke, P., Del, Mar. C,, & Kenardy, J. (2005). Psychological interventions for overweight or obesity. *Cochrane database of systematic reviews.*

Smith, LT. (2007) Support for Healthy Eating and Exercise (SHEE). Colorado, *USA: University of Colorado boulder*.

Steinhardt, M., Bezner, J. and Adams, T. (1999). Outcomes of a Traditional Weight Control Program and a Nondiet Alternative: A One-Year Comparison. *The Journal of Psychology*, 133(5), pp.495-513.

Stuart, R. (1971). A three-dimensional program for the treatment of obesity. *Behaviour Research and Therapy*, 9(3), pp.177-186.

Tapper, K., Shaw, C., Ilsley, J., Hill, A., Bond, F. and Moore, L. (2009). Exploratory randomised controlled trial of a mindfulness-based weight loss intervention for women. *Appetite*, 52(2), pp.396-404.

ter Bogt, N., Milder, I., Bemelmans, W., Beltman, F., Broer, J., Smit, A. and van der Meer, K. (2011). Changes in lifestyle habits after counselling by nurse practitioners: 1-year results of the Groningen Overweight and Lifestyle study. *Public Health Nutr.*, 14(06), pp.995-1000.

Toft, U., Kristoffersen, L., Ladelund, S., Ovesen, L., Lau, C., Borch-Johnsen, K., Pisinger, C. and Jørgensen, T. (2008). The impact of a population-based multi-factorial lifestyle intervention on changes in long-term dietary habits. *Preventive Medicine*, 47(4), pp.378-383.

Turner, S., Thomas, A., Wagner, P. and Moseley, G. (2008). A collaborative approach to wellness: Diet, exercise, and education to impact behavior change. *Journal of the American Academy of Nurse Practitioners*, 20(6), pp.339-344.

Vinkers, C., Adriaanse, M., Kroese, F. and de Ridder, D. (2014). Efficacy of a self-management intervention for weight control in overweight and obese adults: a randomized controlled trial. *Journal of Behavioral Medicine*,37(4),781-792

von Gruenigen, V., Frasure, H., Kavanagh, M., Janata, J., Waggoner, S., Rose, P., Lerner, E. and Courneya, K. (2012). Survivors of uterine cancer empowered by exercise and healthy diet (SUCCEED): A randomized controlled trial. *Gynecologic Oncology*, 125(3), pp.699-704.
